# Development and validation of a structured observation scale to measure responsiveness of physicians in rural Bangladesh

**DOI:** 10.1186/s12913-017-2722-1

**Published:** 2017-11-21

**Authors:** Taufique Joarder, Ilias Mahmud, Malabika Sarker, Asha George, Krishna Dipankar Rao

**Affiliations:** 1 BRAC James P Grant School of Public Health, BRAC University, 68 Shahid Tajuddin Ahmed Sharani, Level 6, icddr,b Building, Dhaka, Mohakhali 1212 Bangladesh; 2 Department of Public Health, College of Public Health and Health Informatics, Qassim University, Bukayriah, Post box: 828, Post code: 51941 Qassim, Saudi Arabia; 3 School of Public Health, University of Western Cape, Cape Town, South Africa; 4 Department of International Health, Johns Hopkins Bloomberg School of Public Health, 615 North Wolfe Street, Suite E8132, Baltimore, MD 20205 USA

**Keywords:** Responsiveness, Human resources for health, Physicians, Psychometrics, Health systems, Bangladesh

## Abstract

**Background:**

Responsiveness of physicians is the social actions that physicians do to meet the legitimate expectations of service seekers. Since there is no such scale, this study aimed at developing one for measuring responsiveness of physicians in rural Bangladesh, by structured observation method.

**Methods:**

Data were collected from Khulna division of Bangladesh, through structured observation of 393 patient-consultations with physicians. The structured observation tool consisted of 64 items, with four Likert type response categories, each anchored with a defined scenario. Inter-rater reliability was assessed by same three raters observing 30 consultations. Data were analyzed by exploratory factor analysis (EFA), followed by assessment of internal consistency by ordinal alpha coefficient, inter-rater reliability by intra-class correlation coefficient (ICC), concurrent validity by correlating responsiveness score with waiting time, and known group validity by comparing public and private sector physicians.

**Results:**

After removing items with more than 50% missing values, 45 items were considered for EFA. Parallel analysis suggested a 5-factor model. Nine items were removed from the list owing to < 0.50 communality, <0.32 loading in un-rotated matrix, and <0.30 on any factor in rotated matrix. Since 34 items (i.e., the number of remaining items after removing nine items by EFA) were loaded neatly under five factors, explained 61.38% of common variance, and demonstrated high internal consistency with coefficient of 0.91, this was adopted as the Responsiveness of Physicians Scale (ROP-Scale). The five factors were named as 1) Friendliness, 2) Respecting, 3) Informing and guiding, 4) Gaining trust, and 5) Financial sensitivity. Inter-rater reliability was high, with an ICC of 0.64 for individual rater’s reliability and 0.84 for average reliability scores. Positive correlation with waiting time (0.51), and higher score of private sector by 0.18 point denote concurrent, and known group validity, respectively.

**Conclusions:**

The ROP-Scale consists of 34 items grouped under five factors. One can apply this with confidence in comparable settings, as this scale demonstrated high internal consistency and inter-rater reliability. More research is needed to test this scale in other settings and with other types of providers.

**Electronic supplementary material:**

The online version of this article (10.1186/s12913-017-2722-1) contains supplementary material, which is available to authorized users.

## Background

Responsiveness of health care providers is an essential attribute of their performance. The concept of responsiveness has appeared in the literature on human resources for health (HRH). In 2004, the Joint Learning Initiative on HRH used the term ‘responsiveness’ in the context of HRH, but did not elaborate further [1]. In 2006, Dieleman and Harnmeijer [2] proposed an analytical framework for HRH performance measurement. This framework suggested four domains of HRH performance, including responsiveness. The World Health Report of 2006 also used the same framework around the same time [3]. However, none of these reports provided any clear definition of HRH responsiveness. Based on literature on responsiveness, patient satisfaction, service quality, doctor-patient communication, as well as relevant studies in other fields (e.g., gender sensitivity, cultural competency) [4], in this paper, we adopted the following definition of HRH responsiveness: “social actions by health providers to meet the legitimate expectations of service seekers”*.*

By the term ‘social action’, actions of health providers related to the therapy or technical aspects of care are excluded; only the non-medical aspects of care are included under HRH responsiveness. The term ‘legitimate expectation’ used in this definition demands explanation. Thompson and Sunol [5] classified expectations as: 1) ideal expectations- clients’ idealistic perception about available services; 2) predicted expectations- clients’ realistic expectations based on experiences, information about available services, etc.; 3) normative expectations- clients’ expectations about what ought to happen; and 4) unformed expectations- clients’ unarticulated expectations (due to various reasons such as lack of understanding, difficulty expressing in language, fear, anxiety, social norms, etc.). De Silva [6] argued, ‘legitimate expectation’ is aligned with the concept of ‘normative expectations’. She defined ‘legitimate’ as, ‘…conforming to recognized principles or accepted rules and standards’ (p. 04), and suggested legitimate expectations be determined based on ethical norms and values.

Responsiveness of HRH, such as physicians, is important as lack of it may dissuade patients from early care seeking, diminish their interest in adopting preventive health information [6–8], and decrease their trust in health service providers [9]. Studies also indicate a discourteous attitude in physicians often compromises care-seeking by specific population groups such as the elderly, patients suffering from non-communicable diseases [10], expectant and new mothers [11], and the lesbian-gay-bisexual-transgender (LGBT) community [12–14], leading to compromised wellbeing.

Responsiveness is also important in Bangladesh health systems context. According to three surveys from 1999, 2000, and 2003, the most important predictor of satisfaction of patients with health providers was found to be the behavior of the providers with the patients [15–17]**.** Dissatisfaction among service seekers over the provider’s behavior has often been expressed in the form of physical violence, as reported by many recent media reports [18–20], as well as by scientific studies [21–23]. Physicians also responded to these acts by holding strikes and refusing services [24–26]. These incidents indicate how important responsiveness of physicians is in the health systems context of countries like Bangladesh.

There are very few studies on the responsiveness of HRH [27–30], especially on physician responsiveness. Among these studies, one primarily focused on HRH performance and responsiveness was discussed as a component of performance, but the psychometric methods of developing the measurement tool was not described [28]. Another study involved telephone interviews in eight European countries, the context of which is much different than Bangladesh [27]. Another study from Brazil described the psychometric steps in developing an instrument to assess the responsiveness of nurses [30]. Another study was from Thailand; and it employed simulated patient method to analyze degree of responsiveness of physicians; but did neither clarify the concept of responsiveness nor investigate the reliability and validity of the tool used [29].

Since responsiveness is shown by service providers and is experienced by service seekers, the data need to come from the actual interaction of both parties. Therefore, in the context of this study, where recording the actual behavior of the physicians is intended, observing the actual interaction, instead of interviewing the clients or providers, can achieve this goal better. In similar studies, different approaches—such as reviewing patients’ records, direct observation of provider, interviews of providers, exit interviews with patients, and simulated patients methods—have been attempted and compared [31–33]. Franko, Daly, Chilongozi, and Dallabetta [32] showed direct observation to be the method of choice (comparing direct observation with provider interviews and simulated patients—in the context of quality of case management of sexually transmitted diseases); however, several studies discussed caveats of this method. For example, service providers may change their behavior when they are aware that they are being observed (Hawthorne effect) [34–36]. But Leonard and Masatu [34] showed in their study that the performance of the observed physicians tend to return to the pre-observation state after the tenth observation. Based on these findings from other studies, we adopted the ‘structured observation’ (SO) method [37], and allowed the first 10 observations to serve as ‘washout’ consultations. We recorded only the eleventh observation in order to avoid or at least minimize the potential Hawthorne effect.

The aim of this study was to develop a scale for measuring responsiveness of physicians in rural Bangladesh. The literature review highlighted the lack of a psychometrically validated scale to measure physician responsiveness in low and middle-income country contexts. By developing such a scale in the context of rural Bangladesh, this paper will add to our understanding of responsiveness and its measurement. Further, it provides a tool which researchers in Bangladesh and other contexts can use to measure health worker responsiveness.

## Methods

A cross-sectional survey of physicians was conducted in Khulna, Bangladesh between December 2014 and January 2015, using an SO checklist.

### Sampling

In this study, we observed consultation sessions of formal sector physicians working either in the public or private sectors. They usually hold a minimum of an MBBS degree (or equivalent foreign degree), and are licensed formally through Bangladesh Medical and Dental Council. The observations were done only in outpatient settings (i.e., consultation rooms) and with the general practitioners. Cases requiring emergency or inpatient care (e.g., assaults, road traffic accidents, poisoning, etc.); or cases requiring additional privacy and confidentiality (e.g., sexually transmitted infections, gynecological conditions, etc.) or physicians’ consultations with children under 18 years were excluded.

A common approach for calculating sample size for factor analysis is five to 10 respondents per item [38–40]. The ratio we adopted was 6:1. Since the initial SO tool consisted of 64 items, we needed a total of 384 physician-consultation observations. However, we sampled 400 physicians to observe their consultations, anticipating unavailability of some physicians during the data collection period (December 2014 and January 2015).

### Recruitment procedure

**Fig. 1:**
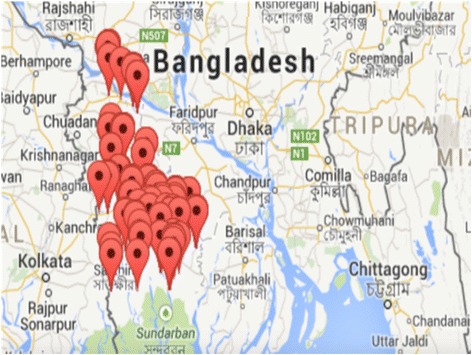
Map of sampled consultations

A list of all physicians who were likely to be present during the data collection period was prepared beforehand. Since most of the physicians were concentrated in and around the Khulna district under Khulna division, we centered in Khulna district and then expanded our field around Khulna district until we reached the desired number (Fig. 1). We chose the census method, as there were no sufficient physicians for sampling. We managed to collect data from 393 consultation sessions (one session per physician) - 195 from public sector and 198 from private sector. The physicians were initially contacted by the first author; then again by the Research Assistant (RA) prior to the observation, i.e., during consent seeking. All but two physicians consented the data collection. The unit of data generation was the observation of consultations; not the individual physicians or the patients per se. Thus, a physician was counted in the public sector if s/he was observed in a public sector setting (e.g., Upazila Health Complex); and private sector if observed in a private sector setting (e.g., clinic, pharmacy, chamber in residence, etc.).

### Measurement model and item generation

The first step of scale development is to determine the unobservable latent variable and the observable indicators or items that would measure the intended latent variable [38]. In this model, the latent variable is responsiveness, which would be measured through 64 observable items or indicators. These items were generated through formative qualitative research, and review of relevant literature [4] (for source of each item, please refer to Additional file 1).

Based on the initial item-pool, an SO tool was developed, with observable response categories (the tool is available as Additional file 2). Each response category was anchored with a scenario. In the SO tool with Likert type responses, response category ‘1’ was the lowest score, which represented a physician lacking responsiveness at all. Scenario for response categories ‘2′ was representative of a typical physician while scenario for ‘3′ was of a better than average responsive physician. Response category ‘4′ was the best practice or a textbook scenario. Items that could not be observed due to inapplicability in the given context or any other reasons were coded as ‘not applicable’. The scenarios for response categories were developed through a qualitative study [4], but category ‘4′ scenarios were mostly taken from text books on clinical practice. The opposite to those were scenario ‘1’s. The middle ones (i.e., ‘2′ and ‘3′) were directly derived from the qualitative data, where patient respondents commented on what they expected from a responsive physician. These scenarios were further calibrated later through inputs from a series of field tests, involving 20 RAs. Their field-based experiential inputs were integrated through group discussions over a period of 10 days. An even number of responses was adopted to avoid choosing the neutral option by raters, which is typically the middle option in an odd-number response pool [38].

### Data collection

The cloud-based mobile software Magpi [41] was used for data collection. The RAs were instructed not to take out the SO tool in front of the physicians. They took notes during the observation and then came out of the room and recorded in their notebook the findings, guided by the hard copy of the SO tool. Then they inputted the data in their phones, uploaded the data, and sent a confirmatory message to the first author.

The RAs recorded the observation of only the 11th patient (allowed the first 10 patients as ‘washout’ observations, in order to minimize Hawthorne effect by the observed physicians), came out of the consultation room with the patient and asked the patient some background information (age, gender, and education). RAs were recommended to observe two consultations per day; but they were strictly instructed not to observe more than three in a day, as large number of observations in a day might diminish data quality.

For the inter-rater reliability test, the first author—along with two RAs—collected the data. The data collection procedure was the same as before, but three observers did the observation simultaneously, but uploaded the data separately. Thirty consultations—15 in the public sector and 15 in the private sector– were observed.

### Statistical analysis

Data collected through Magpi software were imported into Stata version 12.1 for data management, cleaning, missing value imputation, and descriptive analyses [42]. Items with more than 50% non-response or missing values were dropped (shown in Additional file 1, in italicized font), and the remaining missing values in the dataset were imputed by ‘hotdeck’ method [43]. Univariate and multivariate analyses of remaining items were preformed to examine skewness and kurtosis, in order to check the suitability for using polychoric correlations. Skewness or kurtosis of any item greater than one in absolute value in univariate analysis; or a statistically significant skewness or kurtosis in multivariate test support the use of polychoric correlation matrix [44].

Exploratory factor analysis (EFA) was conducted using an open-source software, FACTOR version 9.3.1 [45]. Polychoric correlation matrix was used for the purpose, which is suitable for scales with ordinal response categories [46–48]. The software FACTOR performs the check of suitability of data for factor analysis by Bartlett’s test and Kaiser-Meyer-Olkin (KMO) test. A statistically significant Bartlett’s test and >0.80 KMO statistic indicate the data-suitability for EFA [44]. We chose the minimum rank factor analysis (MRFA) as extraction method [49–51], and for deciding the number of factors to be extracted, adopted the variant of parallel analysis based on MRFA, which is suitable for categorical variables [49]. Factors were rotated using Promin oblique rotation method [46].

After EFA, the model was checked for internal consistency, using the ordinal alpha coefficient, based on polychoric correlation matrix [50], using statistical software R, version 3.1.3 [51]. The corrected item-total correlation was also calculated with a hope to achieve a correlation over 0.35 [39].

For optimizing scale length by dropping items, following three criteria were used: 1) items with communality <0.50; 2) loading of <0.32 of an item on any of the un-rotated factors; and 3) loading of <0.30 (a default value set by the software FACTOR) of an item on any of the rotated factors. Several factor solutions were examined and the 5–factor solution was retained because adding or removing an extra factor could not improve the model in any way (increasing the communality of the items, and/or increasing the loading of items). After three iterations, nine items were dropped and the 34-item model was considered final.

Finally, the ordinal alpha coefficient was assessed to see if dropping an item would increase the alpha coefficient and increase the internal consistency of the model. Since no such item was found, we finalized the 34-item scale, grouped under five factors or subscales. We ran the whole EFA again and found the model optimum and adequate (no item with low communality, each item sufficiently loaded on one factor, high alpha coefficient).

The responsiveness scale score was measured as the mean of the 34 items’ scores. Since this is a continuous value, inter-rater reliability was measured using intra-class correlation coefficient (ICC) [52]. We employed three same raters to rate all the consultations (30 consultations each), and ICC (2, 1) and (2, 3) was calculated. A value of ICC less than 0.40 is considered poor, between 0.40 and 0.59 is fair, between 0.60 and 0.74 is good, and between 0.75 and 1.00 is excellent [53]. We hoped to achieve a correlation value of 0.60 or higher (i.e., good inter-rater reliability).

Criterion validity of the newly developed Responsiveness of Physicians Scale (ROP-Scale) was assessed examining concurrent validity of the scale and known group validation. To investigate concurrent validity, Pearson correlation test was used; and two-sample *t*-test was used for known group validation. For investigating concurrent validity, correlation between ROP-Scale score and consultation time was assessed under the assumption that, responsiveness would be positively correlated with consultation time. Although there is no study establishing this relationship directly, there are studies showing that patients expect more time from physicians on consultation, and that consultation time is a predictor of satisfaction [54]. A correlation coefficient of 0.40 or higher was considered acceptable. For known group validation, the mean responsiveness score of the observations in public sector was compared to that of private sector, under the assumption that physicians in private sector would have statistically significantly higher mean responsiveness score than that in the public [55–57].

## Results

### Background characteristics

#### Items retained for factor analysis

The initial SO tool consisted of 64 items, 19 of which had more than 50% missing values; hence were dropped from any subsequent analyses (Additional file 1). Univariate analysis of the interim scale with 45 variables (i.e., after dropping 19 items) revealed that 21 out of 45 items had skewness or kurtosis greater than one in absolute value. The multivariate test for skewness was not statistically significant, but that for kurtosis was significant with *p*-value <0.01. These suggest using polychoric correlation instead of Pearson’s correlation for factor analysis. Bartlett’s test was statistically significant (with statistic of 6096.1; df of 990 and *p*-value <0.01), and KMO statistic 0.83; both of which indicate the data to be suitable for factor analysis.

### Characteristics of sample

Table 1 summarizes the characteristics of the consultations, physicians, and patients. Half of the observations were done in the public sector and half in the private sector. Average consultation time was five minutes. The majority of the physicians were below 40 years of age and most of them were male. More than half of them had less than two years of experience of working in rural areas. Almost one third of them belonged to the same sub-district where they were observed. Patients were from different age groups, but most of them were females (60%). Almost half of them had less than or equal to primary education, about one third had up to secondary education and the remaining had more than that.

**Table 1:** Characteristics of the consultations, physicians, and patients

Variable		Value
Observation setting	Public sector	195 (n)
	Private sector	198 (n)
Self-reported number of patients seen by physicians in that setting (public or private) per day (Mean and Standard Deviation)		30.35 (17.81)
Consultation time in minutes (mean and standard deviation)		5.04 (2.45)
Gender of physician	Male	78.37 (%)
	Female	21.63 (%)
Age of physician	Less than 30 Years	33.84 (%)
	30 to less than 40 Years	35.62 (%)
	40 to less than 50 Years	11.45 (%)
	More than or equal to 50 years	19.08 (%)
Origin of physician (i.e., whether from the same upazila)	Local	33.33 (%)
	Not local	66.67 (%)
Year of graduation of physician	After 2000	68.18 (%)
	Between 1990 and 2000	11.70 (%)
	Between 1980 and 1990	17.30 (%)
	Before 1980	3.82 (%)
Rural work experience of physician	2 Years or less	51.91 (%)
	More than 2 to 5 years	16.03 (%)
	More than 5 to 10 years	9.92 (%)
	More than 10 years	22.14 (%)
Type of medical college the physician passed from	Public	92.62 (%)
	Private	6.62 (%)
	Foreign	0.76 (%)
Gender of patient	Male	39.69 (%)
	Female	60.31 (%)
Age of patient	Less than 30 years	23.16 (%)
	30 to less than 40 years	20.87 (%)
	40 to less than 50 years	24.94 (%)
	More than or equal to 50 years	31.04 (%)
Level of education of patient	Illiterate	21.88 (%)
	Up to primary (5 Years) education	26.72 (%)
	Up to secondary (10 Years) education	32.06 (%)
	More than secondary education	19.34 (%)

### Factor analysis

#### Determining the number of factors to retain

Parallel analysis suggested the extraction of a 5-factor model. There were five factors whose real data percentage of common variance exceeded the mean or 95 percentile of that of the random datasets generated by the parallel analysis method.

### Factor extraction and rotation

Based on the factor extraction criteria mentioned in the methods section, the following eleven items were dropped from the model: Self identification by doctor, taking consent in general, involving patients in care-related decision making, considering religious and cultural orientation of the patient, legibility of prescription, not showing hierarchical difference, gender sensitivity, interruption during consultation, appearance of doctor, allowing patient to ask questions, and relaxedness and confidence. In the final factor analysis with 34 items and five factors, no item was found to be eligible for being dropped, based on the three criteria mentioned earlier. The remaining items neatly loaded (none of the remaining items had <0.50 communality, <0.32 loading in un-rotated matrix, and <0.30 on any factor in rotated matrix) on five factors, as shown in Table 2.

**Table 2:** Rotated pattern matrix (34 items)

Variable	Friendliness	Gaining trust	Respecting	Informing and guiding	Financial sensitivity
Greetings by doctor	0.34		**0.49**		
Asking patient’s name	**0.42**				
Engaging in social talks	**0.86**				
Asking about patient’s family	**0.85**				
Friendliness	**0.88**				
Showing respect explicitly			**0.69**		
Listening to patient’s complaints completely			**0.84**		
Listening to patient’s complaints attentively			**0.77**		
Examining the patient with care			**0.47**		
Suggestions on disease prevention and health promotion in general				**0.58**	
Giving courage and reassurance	**0.54**				
Earning trust of patients		**0.82**			
Service oriented, not businesslike behavior		**0.87**			
Considering socio-economic status of the patient					**0.91**
Trying to understand socio-economic status of the patient					**0.82**
Informing the cost of treatment/financial counseling				0.31	**0.71**
Providing financial assistance if needed					**0.80**
Facilitating follow-up				**0.35**	
Quantity of issues explained and the quality of explanation				**0.85**	
Quantity of issues explained				**0.84**	
Asking patient if s/he understood the explanation				**0.37**	
Explaining the cause of disease to the patient				**0.81**	
Explaining the diagnosis of disease to the patient				**0.73**	
Explaining the prognosis of disease to the patient				**0.69**	
Explaining the treatment to the patient				**0.45**	
Explaining the preventive aspects to the patient				**0.62**	
Encouraging patient to ask questions			**0.73**		
Listening attentively to patient’s questions			**0.56**		
Not using jargon		**0.54**			
Closing salutation by doctor	0.33		**0.49**		
Non-verbal communication by doctor			**0.68**		
Compassionately touching the patient by doctor		−0.36	**0.55**		
Not being involved in illegal activities		**0.70**			
Sense of humor	**0.76**				

Note**:** Items that are finally retained in the scale under the factors are shown in bold font

The items ‘Greetings by doctor’ and ‘Closing salutation by doctor’ were also loaded somewhat heavily (with loadings of 0.34 and 0.33 respectively) on ‘Friendliness’ factor. But, since their loading was slightly higher in the ‘Respecting’ domain, they are placed under that domain.

In this model, the KMO statistic improved further to be 0.84, and it explained 61.38% of common variance. The highest two inter-factor correlations were between factors three and four (Respecting and Informing and guiding) and factors one and three (Friendliness and Respecting) (Table 3). These correlations justify the use of an oblique factor rotation method instead of an orthogonal method. These high correlations also indicate that some items under the domain ‘Respecting’ can also be seen as a gesture of friendliness and aptitude of the physician in informing and guiding the patient.

**Table 3:** Inter-factor correlation matrix (34 items)

Factor	Friendliness	Gaining trust	Respecting	Informing and guiding	Financial sensitivity
Friendliness	1.00				
Gaining trust	−0.09	1.00			
Respecting	0.42	0.24	1.00		
Informing and guiding	0.40	−0.04	0.43	1.00	
Financial sensitivity	0.25	0.11	0.22	0.25	1.00

Since the scale is intended to measure the responsiveness of physicians, it has been named as the Responsiveness of Physicians Scale, or in short ROP-Scale. The scale is composed of five sub-scales: 1) Friendliness (with items such as asking patient’s name, engaging in social talks, etc.), 2) Gaining trust (with items such as earning trust of patients, not being involved in illegal activities, etc.), 3) Respecting (with items such as showing respect explicitly, listening to patient’s complaints completely, etc.), 4) Informing and guiding (with items such as explaining the cause of disease to the patient, explaining the diagnosis of disease to the patient, etc.), and 5) Financial sensitivity (with items such as considering socio-economic status of the patient, informing the cost of treatment, etc.). The final ROP-Scale, along with the definition of the sub-scales and associated items, has been shown in Table 4.

**Table 4:** The Responsiveness of Physicians Scale (ROP-Scale)

Name of Factor	Definition	Items in domain
Friendliness	How a physician communicates with a patient	1. Asking patient’s name 2. Engaging in social talks 3. Asking about patient’s family 4. Friendliness 5. Giving courage and reassurance 6. Sense of humor
Respecting	How a physician explicitly shows respect to a patient	7. Greetings by doctor 8. Showing respect explicitly 9. Listening to patient’s complaints completely 10. Listening to patient’s complaints attentively 11. Examining the patient with care 12. Encouraging patient to ask questions 13. Listening attentively to patient’s questions 14. Closing salutation by doctor 15. Non-verbal communication by doctor 16. Compassionately touching the patient by doctor
Informing and guiding	How a physician empowers a patient	17. Suggestions on disease prevention and health promotion in general 18. Facilitating follow-up 19. Quantity of issues explained and the quality of explanation 20. Quantity of issues explained 21. Asking patient if s/he understood the explanation 22. Explaining the cause of disease to the patient 23. Explaining the diagnosis of disease to the patient 24. Explaining the prognosis of disease to the patient 25. Explaining the treatment to the patient 26. Explaining the preventive aspects to the patient
Gaining trust	How a physician may gain trust of the patients, or refrains from doing something that may breach trust of the patients	27. Earning trust of patients 28. Service oriented, not businesslike behavior 29. Not using jargon 30. Not being involved in illegal activities
Financial sensitivity	Understanding financial need of the patients and providing support if needed, going beyond the consultation	31. Considering socio-economic status of the patient 32. Trying to understand socio-economic status of the patient 33. Informing the cost of treatment 34. Providing financial assistance if needed

To measure the aggregated ROP-Scale score, the mean of the 34 items was calculated. Subscale scores were calculated in the same way. The mean responsiveness score and subscale scores of the whole sample as well as the sample disaggregated by their sectoral affiliation (i.e., public and private sector) has been shown in Table 5.

**Table 5:** Responsiveness score of the sample using ROP-Scale

Scale	Overall mean score (*n* = 393)	Public sector mean score (*n* = 195)	Private sector mean score (*n* = 198)
Friendliness	1.49 (0.48)	1.34 (0.43)	1.64 (0.48)
Respecting	2.37 (0.41)	2.22 (0.39)	2.51 (0.38)
Informing and guiding	1.80 (0.45)	1.68 (0.46)	1.91 (0.40)
Gaining trust	3.38 (0.37)	3.45 (0.28)	3.32 (0.43)
Financial sensitivity	1.58 (0.59)	1.65 (0.64)	1.51 (0.53)
ROP-Scale	2.07 (0.31)	1.98 (0.29)	2.16 (0.29)

Note: Standard deviation is shown in the parenthesis

### Scale reliability and validity

#### Reliability

The internal consistency of the whole scale was high with an alpha value of 0.91. The alpha value for subscales Friendliness, Gaining trust, Respecting, Informing and guiding, and Financial sensitivity were 0.86, 0.77, 0.87, 0.86, and 0.84, respectively.

Corrected item-total correlations of most of the items were also high in the overall responsiveness scale, ranging from 0.21 to 0.65, with the exception of two items—Not using jargon and Not being involved in illegal activities. However, in respective subscales, these items had high corrected item-total correlations (0.41 and 0.48 respectively).

In order to measure inter-rater reliability, ICC was counted. ICC (2, 1) or individual rater’s reliability score was 0.64 (95% confidence interval 0.37, 0.81), while ICC (2, 3) or average reliability score for three raters was 0.84 (95% confidence interval 0.64, 0.93).

#### Validity

We found a positive correlation of 0.51 between responsiveness score and consultation time, which indicates acceptable concurrent validity of the ROP-Scale. The two sample *t*-tests for the difference in mean responsiveness score revealed that the private sector physicians had significantly higher responsiveness of 0.18 points (*p*-value <0.01) (Table 5)—denoting the known-group validity of ROP-Scale.

## Discussion and conclusions

Our study contributed to the development of the ROP-Scale, with 34 items, grouped under five subscales: Friendliness, Respecting, Informing and guiding, Gaining trust, and Financial sensitivity. These domains and most of the items under each domain are consistent with the relevant studies in this regard (Complete list of items that are aligned with different articles, is available in Appendix 12 of Joarder, 2015 [4]). The scale was found to be reliable, valid, and internally consistent. Another important feature of this study was the use of the same three raters to evaluate inter-rater reliability. This method of calculating ICC is considered useful, as in this method systematic bias between raters is controlled [58].

We found that some items of ‘Friendliness’ domain (e.g., ‘Greetings by doctor’ and ‘Closing salutation by doctor’) were also loaded in the ‘Respecting’ domain. An explanation of this may be, exchanging greeting words or closing salutation are generally out of therapeutic culture of Bangladeshi physicians [59]. Therefore, if a physician does these, the patients see it as a display of respect rather than a display of just friendliness.

In ‘Respecting’ domain, items like ‘Non-verbal communication by doctor’ and ‘Compassionately touching the patient by doctor’ could arguably be seen as gestures of friendliness. However, in Bangladeshi social context, there is a large power differential, especially in rural areas, between the patients and the physicians [59]. While most of the patients’ education falls below the secondary education, the physicians’ level of education and social position were very high in comparison. So, there may be a generalized lack of friendliness from physicians [60]. As a result, some friendly gestures like head-nodding or touching the patients were perceived by the patients as a rather respectful demeanor by the physicians.

Most of the items in the ‘Informing and guiding’ domain are related to providing explanation by the physicians of different aspects related to the disease or condition. Aujoulat, d’Hoore, and Deccache [61] posited that provision of information should be done in a continuous manner, which can be achieved by regular follow-ups. Their suggestions are congruent with this domain, as this domain consists of an item ‘Facilitating follow-up’ along with the explanation-related items.

Trust, in the context of this research, was conceived as patients’ belief that the physicians would act in the best interest of the patients, not in their own interest [9]. Items loaded in the domain ‘Gaining trust’ are in alignment with this definition, except one item: ‘Not using jargon’. An explanation to this item’s loading under ‘Gaining trust’ domain may be using too much technical vocabulary by physicians may depict them in an untrustworthy light. Another feature of this domain is the inclusion of the item ‘Not being involved in illegal activities’, which is supported by previous studies in Bangladesh [17, 56, 59, 62–64]. However, in countries or settings where vigilance or monitoring of the physicians is more scrupulous, or where accountability mechanisms for physicians are better functioning, this item may not seem as appropriate.

The final domain is ‘Financial sensitivity,’ which entails items related to understanding financial status of the patients by doctors and providing support if necessary. A noteworthy feature of this domain is that, most of the items under this domain were derived from the formative qualitative research [4], not from the literature review. The only item that is supported by literature is ‘Informing the cost of treatment’ [65, 66]. But interestingly, according to the formative qualitative research [4], physicians in Bangladesh do not consider providing this type of information as their responsibility. Another item ‘Providing financial assistance if needed’ may be outside of the responsibility of the physicians in settings where pre-payment-based health financing mechanism is established and out-of-pocket payment is uncommon.

It is clear from the above discussion that, while some items of the ROP-Scale are commonly found in other literature, few others are very much context specific, i.e., peculiar to Bangladesh or similar settings. Therefore, caution needs to be maintained in generalizing these items to different settings such as western, or advanced industrialized societies. The scale also needs to be carefully validated for measuring responsiveness of other health workers such as the nurses, community health workers (CHW), etc.

### Strengths and limitations of the study

Despite taking careful measures to ensure psychometric rigor, this research may face some criticisms, which are common for most psychometric scales. Major criticism could fall on the decision rules adopted at different decision points. Using a different decision rule or a different method may bring forth a different model. So, we first tried to ensure face and content validity of the items through repeated consultations with the experts who have reasonable expertise on the subject matter and/or the context of where and among whom the study was conducted [4]. Significant efforts were put in repeated field-tests too.

Criterion (concurrent) validity could not be ascertained properly due to the lack of a gold standard to compare the findings with. Construct validity also could not be assessed. A multi-method approach could be employed for checking construct validity; for example, a separate exit interview tool could have been developed for this purpose. This was not done due to time and resource limitations. Test-retest reliability could not be assessed due to the methodological limitation. As the consultation scenario changes from patient to patient, test-retest reliability was not possible to measure, given the methods adopted for this study (i.e., SO method). However, this could be attempted if an exit interview method was used.

Finally, we acknowledge the fact that separating the ‘medical’ or ‘technical’ aspects of care from the ‘non-medical’ or ‘social’ aspects is not straightforward, as many ‘social’ actions may have implications for ‘medical’ aspects of care. For example, one of ROP-Scale items, ‘Examining the patient with care’, despite being included here as a ‘social action’, has clear ‘medical’ values. Similarly, many ‘medical’ actions would render the physician ‘responsive’ in the eyes of the patients. For example, physicians would touch the patients for various therapeutic purposes, which may be considered by patients as a ‘social’ action’ (e.g., Compassionately touching the patient by doctor’).

### Future research

The known-group validation in this study, involving investigation of physicians’ responsiveness in public and private sector, indicates that there might be difference in the level of responsiveness in these two settings. It may be useful to examine the differences in responsiveness between public and private sector physicians more in-depth. It can also be seen if they differ in terms of all the domains of responsiveness, or they differ only in certain domains.

This study was limited to the physicians working in the outpatients of rural areas of Bangladesh. Future studies can be carried out in various other relevant settings such as in the urban areas, among other professional groups like the nurses, CHWs, etc., in other professional settings like inpatient services, emergency, etc.

This study focused on developing the responsiveness scale, but this did not take into account many potential determinants of responsiveness, which may aid the physicians to be responsive or deter them from being responsive in practice. Understanding of these determinants is crucial to improve the responsiveness and resolve the issues around this topic.

### Policy implications

Since measuring the magnitude of a problem is one of the crucial steps of public health problem solving paradigm [67], this scale can contribute in this regard and assist the policy makers to understand the absolute magnitude (overall responsiveness score), relative magnitude (domain-specific responsiveness score) and distribution (responsiveness score across geographical areas, professional groups, etc.) of the deficiencies in this front.

As performance based payment and other modalities of result based financing mechanism are gaining popularity, public health managers or program implementers would need to measure responsiveness as a part of the performance of HRH. The ROP-Scale can help in evaluating and monitoring HRH performance; hence it has the potential to be utilized in a performance based payment scheme.

Although our study was done in rural Bangladeshi setting, this may provide conceptual and methodological inputs to conduct similar locally relevant studies in other countries. Series of such studies may aid in developing a tool, robust enough to conduct cross-national comparisons, at least in comparable countries.

## Additional files

Additional file 1: List and sources of all items in quantitative structured observation tool. (DOCX 112 kb)

Additional file 2: Structured observation tool (full version-64 items). (DOCX 177 kb)

## Abbreviations

BM&DC: Bangladesh Medical and Dental Council
CHW: Community Health Worker
EFA: Exploratory Factor Analysis
HRH: Human Resources for Health
ICC: Intra-class Correlation Coefficient
JHSPH: Johns Hopkins School of Public Health
JPGSPH: James P Grant School of Public Health
KMO: Kaiser-Meyer-Olkin
MRFA: Minimum Rank Factor Analysis
RA: Research Assistant
ROP-Scale: Responsiveness of Physicians Scale
SO: Structured Observation
