# Supplementary material for: Development and validation of a structured observation scale to measure responsiveness of physicians in rural Bangladesh
Source: BMC Health Serv Res. 2017 Nov 21;17:753. doi: 10.1186/s12913-017-2722-1 (PMC5697080; doi:10.1186/s12913-017-2722-1)
Supplement: Supplementary file 1 — List and sources of all items in quantitative structured observation tool. (DOCX 112 kb) [file 12913_2017_2722_MOESM1_ESM.docx]

## Additional file 1: List and sources of all items in quantitative structured observation tool

| **Variable Number** | **Item** | **Item number in the final scale** | **Source: formative (qualitative) research** | **Source: literature review** | **Reference** |
| --- | --- | --- | --- | --- | --- |
| 1 | **Greetings by doctor** | 7 | √ | √ | Fassaert et al., 2007; Makoul, 2001 |
| 2 | *Response of doctor to patient's greetings* |  | √ |  |  |
| 3 | Self Identification by doctor |  |  | √ | Blanchard et al., 1983; Rodriguez et al., 2012 |
| 4 | **Asking patient's name** | 1 | √ | √ | Abdulhadi et al., 2006; Carey & Seibert, 1993; Forouzan et al., 2011; Rodriguez et al., 2012; Wolf et al., 1978) |
| 5 | **Engaging in social talks** | 2 | √ | √ | Abdulhadi et al., 2006; Beck et al., 2000; Blanchard et al., 1983; Boon & Stewart, 1998; Fassaert et al., 2007; Makoul, 2001; White et al., 1991 |
| 6 | **Asking about patient's family** | 3 | √ | √ | Blanchard et al., 1983 |
| 7 | **Friendliness** | 4 | √ | √ | Abdulhsadi et al., 2006; Beck et al., 2000; Walbridge & Delene, 1993; Wolf et al., 1978 |
| 8 | **Showing respect explicitly** | 8 | √ | √ | Beck et al., 2000; Carey & Seibert, 1993; Darby et al., 2000; DeSilva, 1999; Elwyn et al., 2005; Forouzan et al., 2011; Hsu et al., 2006; Letkovicova et al., 2005; Rao et al., 2006; Stewart, 1984 |
| 9 | **Listening to patient's complaints completely** | 9 | √ | √ | Fassaert et al., 2007; Rao et al., 2006; Wolf et al., 1978 |
| 10 | **Listening to patient's complaints attentively** | 10 | √ | √ | Abdulhadi et al., 2006; Beck et al., 2000; Coulter & Jenkinson, 2005; Fassaert et al., 2007; Forouzan et al., 2011; Hsu et al., 2006; Letkovicova et al., 2005; Rao et al., 2006 |
| 11 | *Counseling on social or family issues if related to the disease* |  | √ |  |  |
| 12 | *Home visit by doctor* |  | √ |  |  |
| 13 | **Examining the patient with care** | 11 | √ | √ | White et al., 1991 |
| 14 | *Taking consent in particularly necessary conditions* |  | √ |  |  |
| 15 | Taking consent in general |  |  | √ | DeSilva, 1999; Letkovicova et al., 2005 |
| 16 | *Maintaining confidentiality of information* |  | √ | √ | DeSilva, 1999; Letkovicova et al., 2005 |
| 17 | **Suggestions on disease prevention and health promotion in general** | 17 | √ | √ | Abdulhadi et al., 2006; Beck et al., 2000; Darby et al., 2000; Makoul, 2001; Rao et al., 2006; Rice et al., 2008a; Sirven et al., 2008; Valentine et al., 2007 |
| 18 | *Referral practice* |  | √ | √ | White et al., 1991 |
| 19 | *Consultation with colleagues if in confusion* |  | √ |  |  |
| 20 | *Allowing patients to choose doctors* |  | √ | √ | Coulter & Jenkinson, 2005; Darby et al., 2000; DeSilva, 1999; Forouzan et al., 2011; Letkovicova et al., 2005; Murray et al., 2001; Nigel Rice et al., 2008; Valentine et al., 2007; WHO, 2000 |
| 21 | **Giving courage and reassurance** | 5 | √ | √ | Abdulhadi et al., 2006; Beck et al., 2000; Forouzan et al., 2011; White et al., 1997 |
| 22 | **Earning trust of patients** | 27 | √ | √ | Blanchard et al., 1983; Makoul, 2001; Wolf et al., 1978 |
| 23 | **Service oriented, not businesslike behavior** | 28 | √ | √ | White et al., 1997 |
| 24 | *Not sending patients to specific diagnostic centers* |  | √ |  |  |
| 25 | Involving patients in care related decision making |  |  | √ | Boon & Stewart, 1998; Coulter & Jenkinson, 2005; DeSilva, 1999; Forouzan et al., 2011; Letkovicova et al., 2005; Makoul, 2001; Murray et al., 2001; Nigel Rice et al., 2008; Sirven et al., 2008; Valentine et al., 2007; WHO, 2000 |
| 26 | *Considering individual need of the patient* |  | √ | √ | Forouzan et al., 2011; Hsu et al., 2006; Levinson et al., 2008; Makoul, 2001; Walbridge & Delene, 1993 |
| 27 | Considering religious and cultural orientation of the patient |  | √ | √ | Fernandez et al., 2004; Thom & Tirado, 2006; Werkmeister-Rozas & Klein, 2009 |
| 28 | *Facilitating utilization of local resources* |  | √ | √ | Forouzan et al., 2011; Thom & Tirado, 2006 |
| 29 | **Considering socio-economic status of the patient** | 31 | √ |  |  |
| 30 | **Trying to understand socio-economic status of the patient** | 32 | √ |  |  |
| 31 | **Informing the cost of treatment/ financial counseling** | 33 | √ | √ | Walbridge & Delene, 1993; Wolf et al., 1978 |
| 32 | **Providing financial assistance if needed** | 34 | √ |  |  |
| 33 | **Facilitating follow-up** | 18 | √ | √ | Forouzan et al., 2011; Schirmer et al., 2005; Walbridge & Delene, 1993; White et al., 1991 |
| 34 | **Quantity of issues explained and the quality of explanation** | 19 | √ | √ | Beck et al., 2000; Wolf et al., 1978 |
| 35 | **Quantity of issues explained** | 20 | √ | √ | Boon & Stewart, 1998; Rao et al., 2006 |
| 36 | *Explaining everything to the patient by the doctor himself* |  | √ |  |  |
| 37 | **Asking patient if s/he understood the explanation** | 21 | √ | √ | Abdulhadi et al., 2006; Coulter & Jenkinson, 2005; Forouzan et al., 2011; Letkovicova et al., 2005; Thom & Tirado, 2006; White et al., 1997; Wolf et al., 1978 |
| 38 | **Explaining the cause of disease to the patient** | 22 | √ |  |  |
| 39 | **Explaining the diagnosis of disease to the patient** | 23 | √ | √ | Carey & Seibert, 1993; Morphet et al., 2012 |
| 40 | **Explaining the prognosis of disease to the patient** | 24 | √ | √ | Blanchard et al., 1983; White et al., 1997; Wolf et al., 1978 |
| 41 | **Explaining the treatment to the patient** | 25 | √ | √ | Abdulhadi et al., 2006; Blanchard et al., 1983; Carey & Seibert, 1993; Mashego & Peltzer, 2005; Rao et al., 2006; White et al., 1997; Wolf et al., 1978 |
| 42 | **Explaining the preventive aspects to the patient** | 26 | √ | √ | White et al., 1997 |
| 43 | *Explaining the side effects of the treatment to the patient* |  | √ | √ | Beck et al., 2000; Makoul, 2001 |
| 44 | *Explaining the result of tests to the patient* |  | √ | √ | Blanchard et al., 1983; White et al., 1997; Wolf et al., 1978 |
| 45 | Allowing patient to ask questions |  | √ | √ | Beck et al., 2000; DeSilva, 1999; Letkovicova et al., 2005; Rao et al., 2006 |
| 46 | *Answering patient's questions by doctor her/himself* |  | √ |  |  |
| 47 | *Keeping patience in patient's irrelevant questions* |  | √ |  |  |
| 48 | **Encouraging patient to ask questions** | 12 | √ | √ | Abdulhadi et al., 2006; Beck et al., 2000 |
| 49 | **Listening attentively to patient's questions** | 13 | √ | √ | Beck et al., 2000 |
| 50 | **Not using jargon** | 29 | √ | √ | Beck et al., 2000; Bernhart et al., 1999; Fassaert et al., 2007; Fernandez et al., 2004; Makoul, 2001; Wolf et al., 1978 |
| 51 | *Communicating limitations to the patient at the outset* |  | √ |  |  |
| 52 | **Closing salutation by doctor** | 14 | √ | √ | Abdulhadi et al., 2006; Beck et al., 2000; White et al., 1991; White et al., 1997 |
| 53 | *Responding to patient's closing salutation* |  | √ |  |  |
| 54 | Legibility of prescription |  | √ |  |  |
| 55 | Not showing hierarchical difference |  | √ | √ | Forouzan et al., 2011 |
| 56 | Gender sensitivity |  | √ | √ | Berlan & Shiffman, 2012; Haaland & Vlassoff, 2001 |
| 57 | Interruption during consultation |  | √ | √ | Beck et al., 2000; Fassaert et al., 2007; Makoul, 2001; Rhoades, McFarland, Finch, & Johnson, 2001 |
| 58 | Appearance of doctor |  | √ | √ | Andaleeb, 2001; Walbridge & Delene, 1993 |
| 59 | *Establishing discipline in consultation room* |  | √ | √ | Andaleeb, 2001 |
| 60 | **Non-verbal communication by doctor** | 15 | √ | √ | Abdulhadi et al., 2006; Beck et al., 2000; Boon & Stewart, 1998; Fassaert et al., 2007 |
| 61 | **Compassionately touching the patient by doctor** | 16 | √ | √ | Beck et al., 2000; Blanchard et al., 1983 |
| 62 | **Not being involved in illegal activities** | 30 | √ | √ | Andaleeb, 2001 |
| 63 | **Sense of humor** | 6 | √ | √ | Beck et al., 2000; Hojat et al., 2002; White et al., 1997 |
| 64 | Relaxedness and confidence |  |  | √ | Fassaert et al., 2007; Stewart, 1984 |

Italicized items (n = 19) were dropped for having more than 50% non-response or missing values

Bold font items (n = 34) are included in the final version of the item

**Bibliography:**

Abdulhadi, N., Al-Shafaee, M. A., Ostenson, C.-G., Vernby, A., & Wahlström, R. (2006). Quality of interaction between primary health-care providers and patients with type 2 diabetes in Muscat, Oman: an observational study. *BMC Family Practice*, *7*, 72. https://doi.org/10.1186/1471-2296-7-72

Andaleeb, S. S. (2001). Service quality perceptions and patient satisfaction: a study of hospitals in a developing country. *Social Science & Medicine*, *52*(9), 1359–1370. https://doi.org/10.1016/S0277-9536(00)00235-5

Beck, R. S., Daughtridge, R., & Sloane, P. D. (2000). Physician-patient communication in the primary care office: a systematic review. *The Journal of the American Board of Family Practice / American Board of Family Practice*, *15*(1), 25–38.

Berlan, D., & Shiffman, J. (2012). Holding health providers in developing countries accountable to consumers: a synthesis of relevant scholarship. *Health Policy and Planning*, *27*(4), 271–80. https://doi.org/10.1093/heapol/czr036

Bernhart, M. H., Wiadnyana, I. G., Wihardjo, H., & Pohan, I. (1999). Patient satisfaction in developing countries. *Social Science & Medicine (1982)*, *48*(8), 989–96. Retrieved from http://www.ncbi.nlm.nih.gov/pubmed/10390039

Blanchard, C. G., Ruckdeschel, J. C., Blanchard, E. B., Arena, J. G., Saunders, N. L., & Malloy, E. D. (1983). Interactions between oncologists and patients during rounds. *Annals of Internal Medicine*, *99*(5), 694–699.

Boon, H., & Stewart, M. (1998). Patient-physician communication assessment instruments:1986 to 1996 in review. *Patient Education and Counseling*, *35*(3), 161–176. https://doi.org/10.1016/S0738-3991(98)00063-9

Carey, R. G., & Seibert, J. H. (1993). A patient survey system to measure quality improvement: questionnaire reliability and validity. *Medical Care*, *31*(9), 834–845.

Coulter, A., & Jenkinson, C. (2005). European patients’ views on the responsiveness of health systems and healthcare providers. *European Journal of Public Health*, *15*(4), 355–60. https://doi.org/10.1093/eurpub/cki004

Darby, C., Valentine, N., Murray, C. J. L., & De Silva, A. (2000). *World Health Organization: Strategy on measuring responsiveness*. *The Journal of medicine and philosophy* (Vol. 39). Geneva. Retrieved from http://www.who.int/healthinfo/paper23.pdf

De Silva, A. (1999). *A framework for measuring responsiveness*. Geneva. Retrieved from http://www.who.int/healthinfo/paper32.pdf

Elwyn, G., Hutchings, H., Edwards, A., Rapport, F., Wensing, M., Cheung, W. Y., & Grol, R. (2005). The OPTION scale: Measuring the extent that clinicians involve patients in decision-making tasks. *Health Expectations*, *8*(1), 34–42. https://doi.org/10.1111/j.1369-7625.2004.00311.x

Fassaert, T., van Dulmen, S., Schellevis, F., & Bensing, J. (2007). Active listening in medical consultations: Development of the Active Listening Observation Scale (ALOS-global). *Patient Education and Counseling*, *68*(3), 258–264. https://doi.org/10.1016/j.pec.2007.06.011

Fernandez, A., Schillinger, D., Grumbach, K., Rosenthal, A., Stewart, A. L., Wang, F., & Pérez-Stable, E. J. (2004). Physician language ability and cultural competence. *Journal of General Internal Medicine*, *19*(2), 167–174. https://doi.org/10.1111/j.1525-1497.2004.30266.x

Forouzan, A. S., Ghazinour, M., Dejman, M., Rafeiey, H., & San Sebastian, M. (2011). Testing the WHO responsiveness concept in the Iranian mental healthcare system: a qualitative study of service users. *BMC Health Services Research*, *11*(1), 325. https://doi.org/10.1186/1472-6963-11-325

Haaland, A., & Vlassoff, C. (2001). Introducing Health Workers for Change : from transformation threory to health systems in developing countries, *16*, 1–6.

Hojat, M., Gonnella, J. S., Nasca, T. J., Mangione, S., Vergare, M., & Magee, M. (2002). Physician empathy: Definition, components, measurement, and relationship to gender and specialty. *American Journal of Psychiatry*, *159*(9), 1563–1569. https://doi.org/10.1176/appi.ajp.159.9.1563

Hsu, C.-C., Chen, L., Hu, Y.-W., Yip, W., & Shu, C.-C. (2006). The dimensions of responsiveness of a health system: a Taiwanese perspective. *BMC Public Health*, *6*, 72. https://doi.org/10.1186/1471-2458-6-72

Letkovicova, H., Prasad, A., Vallée, R. La, Valentine, N., Adhikari, P., & Heide, G. W. van der. (2005). *The health systems responsiveness analytical guidelines for surveys in the multi-country survey study*. Geneva. Retrieved from http://www.who.int/responsiveness/papers/MCSS_Analytical_Guidelines.pdf

Levinson, W., Hudak, P. L., Feldman, J. J., Frankel, R. M., Kuby, A., & Bereknyei, S. (2008). “It’s Not What You Say . . .,” *46*(4), 10–16.

Makoul, G. (2001). The SEGUE Framework for teaching and assessing communication skills. *Patient Education and Counseling*, *45*(1), 23–34. https://doi.org/10.1016/S0738-3991(01)00136-7

Mashego, T.-A., & Peltzer, K. (2005). Community perception of quality of (primary) health care services in a rural area of Limpopo Province, South Africa: a qualitative study. *Curationis*, *28*(2), 13–21. Retrieved from http://www.ncbi.nlm.nih.gov/pubmed/16045107

Morphet, J., Innes, K., Munro, I., O’Brien, A., Gaskin, C. J., Reed, F., & Kudinoff, T. (2012). Managing people with mental health presentations in emergency departments--a service exploration of the issues surrounding responsiveness from a mental health care consumer and carer perspective. *Australasian Emergency Nursing Journal : AENJ*, *15*(3), 148–55. https://doi.org/10.1016/j.aenj.2012.05.003

Murray, C. J. L., Kawabata, K., & Valentine, N. (2001). People’s Experience Versus People’s Expectations. *Health Affairs*, *20*(3), 21–24. https://doi.org/10.1377/hlthaff.20.3.21

Rao, K. D., Peters, D. H., & Bandeen-Roche, K. (2006). Towards patient-centered health services in India--a scale to measure patient perceptions of quality. *International Journal for Quality in Health Care : Journal of the International Society for Quality in Health Care / ISQua*, *18*(6), 414–421. https://doi.org/10.1093/intqhc/mzl049

Rhoades, D. R., McFarland, K. F., Finch, W. H., & Johnson, A. O. (2001). Speaking and intrruptions during primary care office visits. *Family Medicine*, *33*(7), 528–532.

Rice, N., Robone, S., & Smith, P. C. (2008). *The measurement and comparison of health system responsiveness*. *Health, Econometrics and Data Group (HEDG) Working Papers*. HEDG, c/o Department of Economics, University of York. Retrieved from http://ideas.repec.org/p/yor/hectdg/08-05.html

Rodriguez, A. V. D., Vituri, D. W., Haddad, M. do C. L., Vannuchi, M. T. O., & Oliveira, W. T. de. (2012). The development of an instrument to assess nursing care responsiveness at a university hospital. *Revista Da Escola de Enfermagem Da USP*, *46*(1), 167–74. Retrieved from http://www.ncbi.nlm.nih.gov/pubmed/22441281

Schirmer, J., Mauksch, L., Lang, F., Marvel, K., Zoopi, K., & Epstein, R. (2005). Assesing Comunication Competence: A review of current tools. *Fam Med*, *37*(3), 184–192.

Sirven, N., Santos-Eggimann, B., & Spagnoli, J. (2008). *Comparability of Health Care Responsiveness in Europe Using Anchoring Vignettes from Survey of Health, Aging and Retirement in Europe*. Institut de recherche et documentation en économie de la santé. Retrieved from http://books.google.com/books?id=OOfePgAACAAJ&pgis=1

Stewart, M. A. (1984). What is a successful doctor-patient interview ? A study of interactions and outcomes. *Social Science & Medicine*, *19*(2), 167–175.

Thom, D. H., & Tirado, M. D. (2006). Development and validation of a patient-reported measure of physician cultural competency. *Medical Care Research and Review : MCRR*, *63*(5), 636–655. https://doi.org/10.1177/1077558706290946

Valentine, N. B., Bonsel, G. J., & Murray, C. J. L. (2007). Measuring quality of health care from the user’s perspective in 41 countries: psychometric properties of WHO’s questions on health systems responsiveness. *Quality of Life Research : An International Journal of Quality of Life Aspects of Treatment, Care and Rehabilitation*, *16*(7), 1107–25. https://doi.org/10.1007/s11136-007-9189-1

Walbridge, S. W., & Delene, L. M. (1993). Measuring physician attitudes of service quality. *Journal of Health Care Marketing*, *13*(1), 6–15.

Werkmeister-Rozas, L., & Klein, W. C. (2009). Cultural Responsiveness in Long-Term-Care Case Management: Moving Beyond Competence. *Care Management Journals*, *10*(1), 2–7. https://doi.org/10.1891/1521-0987.10.1.2

White, D. G., Tiberius, R., Talbot, Y., Schiralli, V., & Rickett, M. (1991). Improving Feedback for Medical Students in a Famnily Medicine Clerkship. *Canadian Family Physician*, *37*, 64–70.

White, J. C., Rosson, C., Christensen, J., Hart, R., & Levinson, W. (1997). Wrapping things up: A qualitative analysis of the closing moments of the medical visit. *Patient Education and Counseling*, *30*(2), 155–165. https://doi.org/10.1016/S0738-3991(96)00962-7

Wolf, M. H., Putnam, S. M., James, S. A., & Stiles, W. B. (1978). The Medical Interview Satisfaction Scale: development of a scale to measure patient perceptions of physician behavior. *Journal of Behavioral Medicine*, *1*(4), 391–401. https://doi.org/10.1007/BF00846695

World Health Organization. (2000). *The World health report 2000 : health systems : improving performance.* Geneva.
