# Supplementary material for: Development and validation of a structured observation scale to measure responsiveness of physicians in rural Bangladesh
Source: BMC Health Serv Res. 2017 Nov 21;17:753. doi: 10.1186/s12913-017-2722-1 (PMC5697080; doi:10.1186/s12913-017-2722-1)
Supplement: Supplementary file 2 — Structured observation tool (full version-64 items). (DOCX 177 kb) [file 12913_2017_2722_MOESM2_ESM.docx]

## Additional file 2: Structured observation tool (full version-64 items)

**Understanding and Measuring Responsiveness of Human Resources for Health in Rural Bangladesh**

**Structured Observation Tool**

**1. General Identification Questions**

**Instruction to the observer:** Fill out these information’s just before starting the interview with the doctor and structured observation. Take the first photo of the consultation room (with patients and the doctor if possible) during this time. Take the second photo after the consultation.

2. Observation ID

3. Observer ID

4. Date of observation

5. Location of observation

6. Observation setting: Public sector/private sector

7. Type of provider (in case of private sector): Exclusively Private/ both public and private but observed in private setting only/ both public and private and observed in both settings

8. Geospatial data (longitude/ latitude) [This is best captured in open space]

9. Starting time of observation

10. Ending time of observation

**11. Questions for the Doctor**

**Instruction to the observer:** Ensure you have obtained the consent from both the patients and the doctor. Greet the doctor; introduce yourself, and record this information.

12. ID of the doctor [Write the name, which will be replaced by a numeric ID later]

13. Age of doctor [in years]

14. Gender

15. Degrees [E.g., MBBS, FCPS (Medicine)]

16. Medical College [E.g., Khulna Medical College]

17. Year of graduation

18. Number of months in practice [Including internship]

19. Number of months working in this upazila

20. Number of months working in rural settings

21. Is the doctor originally from this area? [Yes/ No]

22. How many patients does the doctor attend on an average in a typical day (if the observation is in public sector, doctor should give an estimate of patients attended per day in public sector setting and vice versa)? [Feeds into 89]

**23. Observation Items**

**Instruction to the observer:** Be present while consulting with 11 patients. Do not record first 10 observations. Fill out the tool on the basis of last (11th) observation.

**Exclusion Criteria:**

Do not include the following patients in your observation: Those who are below 18 years, suffering from gynecological diseases, venereal diseases, emergency patients and patients suffering from diseases where it is necessary to examine private parts (i.e., where extra privacy is required).

**Beginning part**

24. **Greetings by doctor**

Patients expect that the doctor will greet and welcome the patient, make the patient feel comfort, accept the patient cordially, reply the patient’s greetings and ask the patient’s well being. Assess the role of the doctor in this regard by 1 to 4 where 1-completely unsatisfactory and 4-completely satisfactory. Possible observations are as follows:

| NA-------1-------2--------3--------4 | | | |
| --- | --- | --- | --- |
| The doctor started writing the prescription without any greeting.  If the patient greeted first, the doctor even did not respond to the greetings of the patient. | The doctor expressed minimum greetings to the patient.  If the patient greeted first, doctor responded to the patient’s greetings indirectly (by shaking head, lifting eyes, shaking hand etc.).  **Following greetings among the traditional greetings in this country might have been included:** asking the name of the patient, asking him to take a seat etc. | The doctor greeted and welcomed the patient; however, it was not enough to make the patient easy or friendly.  If the patient greeted first, doctor gave his answer in words when the patient gave salam.  **Following greetings among the traditional greetings in this country might have been included:**  giving salam to the patient (Saying Adab/Nomoskar to the Hindus and others as per their religion), asking the name of the patient, asking his well beings (how are you/what’s the matter), appropriate salutation like mother, father, brother, sister, sister-in-law, asking to take a seat, exchange smiles etc. | The doctor greeted warmly and welcomed the patient friendly and became easy with him.  If the patient greeted first, doctor cordially answered the patient’s and also exchanged greeting.  **Following greetings among the traditional greetings in this country might have been included:**  giving salam to the patient (Saying Adab/Nomoskar to the Hindus and others as per their religion), asking the name of the patient and calling in his name, asking his well beings (how are you/what’s the matter), appropriate salutation like mother, father, brother, sister, sister-in-law, saluting ‘Babu’ (in case of children), Sweety etc. Asking to take a seat, asking whether he had his breakfast, asking about his residence and profession, smiling at him; to shake hands with him; showing respect to the aged person by standing up etc. |

25. **Response of doctor to patient's greetings**

In our country usually patients give Salam to the doctors in most of the cases. The patients expect that the doctor will start consultation after replying Salam and asking the patient’s well being. Assess the role of the doctor in this regard by 1 to 4 where 1-completely unsatisfactory and 4-completelysatisfactory. Possible observations are as follows: **[Use N/A in case the patient did not greet first.]**

| NA-------1-------2--------3--------4 | | | |
| --- | --- | --- | --- |
| The patient gave Salam/ greetings to the doctor, but he did not reply. | The doctor replied the patient’s greetings by gesture (moving neck, eye expression, moving hand etc.), but did not say anything in words. | The doctor replied in brief in response to patient’s greetings. As for example by moving neck, eye expression and at the same time by saying Olaikum Salam. | The doctor replied the patient’s greetings completely and cordially and also s/he himself exchanged some greetings. |

26. **Self Identification by doctor**

The patients become confused if they do not know the doctor’s identity (especially the doctor’s designation, e.g., Medical Officer, Sub Assistant Community Medical Officer etc. or specialty, e.g., pediatrician, gynecologist etc.). So, it is necessary to make arrangement for showing the identity in any consultation. Assess the role of the doctor in this regard by 1 to 4 where 1-completely unsatisfactory and 4-completelysatisfactory. Possible observations are as follows:

| NA-------1-------2--------3--------4 | | | |
| --- | --- | --- | --- |
| If none of the issues mentioned below is present. | If any of the issues mentioned below is present. | If any two of the following issues are present. | If all of the following three issues are present. |
| **There may be following measures for showing the identity:** The doctor’s name and designation was visibly mentioned outside the consultation room. Inside the room the doctor’s identity was written in front of the doctor or in the nameplate in his body. The doctor introduced himself with the patients before consultation. | | | |

27. **Asking patient's name**

Patients expect that the doctor will ask patient's name at least and will treat the patient as a person. Assess the role of the doctor in this regard by 1 to 4 where 1-completely unsatisfactory and 4-completelysatisfactory. Possible observations are as follows:

| NA-------1-------2--------3--------4 | | | |
| --- | --- | --- | --- |
| The doctor did not consider asking the name of the patient as necessary or just asked the name for writing in the prescription. | The doctor asked the name of the patient but it was not seemed that he listened to the patient by giving importance or attention. | The doctor asked the name of the patient and listened attentively. But did not call her/ him in that name. | The doctor asked the name of the patient, listened attentively and also called her/ him in that name. |

28. **Engaging in social talks**

The patient expects that the doctor will not only listen to his problem but also do some social talks and listen to the patient if he does any social talk. Assess the role of the doctor in this regard by 1 to 4 where 1-completely unsatisfactory and 4-completelysatisfactory. Possible observations are as follows:

| NA-------1-------2--------3--------4 | | | |
| --- | --- | --- | --- |
| The doctor did not do any social talk with patient. Even he stopped the patient when the patient started to do some social talks. | The doctor did minimum social talk or responded when the patient started social talk.  **In this case following social talks might have been included:** Who are there in the family etc. | The doctor did some social talk or responded when the patient started social talk.  **In this case following social talks might have been included:** The patient’s profession, family members etc. | The doctor got involved completely into social talk or if the patient started, he participated satisfactorily into social talks.  **In this case following social talks might have been included:** The patient’s profession, education, children, family members, weather etc. |

29. **Asking about patient's family**

The patient expects that doctor will also ask about his family. Assess the role of the doctor in this regard by 1 to 4 where 1-completely unsatisfactory and 4-completelysatisfactory. Possible observations are as follows:

| NA-------1-------2--------3--------4 | | | |
| --- | --- | --- | --- |
| The doctor did not ask anything about the family of the patient or there was nothing in this regard during his consultation. | The doctor did not want to know anything about the family of the patient but there were some talking regarding this in his advice. That means it was a part of his treatment but not a social talk. | The doctor wanted to know about the family of the patient but not in details. That was not a part of the treatment rather a part of social talk.  **For example:** how many members are there in his family, how many children, are they all well etc. | The doctor wanted to know about the family of the patient in details cordially. That was not a part of the treatment rather a part of social talk.  **For example:** how many members are there in his family, how many children, are they all well etc. |

30. **Friendliness**

Patients expect that the doctor will be friendly. Assess the role of the doctor in this regard by 1 to 4 where 1-completely unsatisfactory and 4-completelysatisfactory. Possible observations are as follows:

| NA-------1-------2--------3--------4 | | | |
| --- | --- | --- | --- |
| The doctor was not friendly. | The doctor was friendly at a minimum level. | The doctor was somewhat friendly. | The doctor was fully friendly. |
| **The example of friendliness may be:** remembering the name and face of the patient and calling him by name (here ‘calling by name’ means calling by name of the patient in a friendly manner); asking or making comment about an event of the patient’s family; praising the patient (about clothing or anything else); asking for an opinion of the patient about anything (weather, politics etc.) | | | |

31. **Showing respect explicitly**

Patients do not expect misbehave rather expect good behavior from the doctor. Assess the role of the doctor in this regard by 1 to 4 where 1-completely unsatisfactory and 4-completelysatisfactory. Possible observations are as follows:

| NA-------1-------2--------3--------4 | | | |
| --- | --- | --- | --- |
| The doctor misbehaved with the patient. | The doctor neither misbehaved nor behaved well with the patient. | The doctor showed least but not much respect. **As for example**: replying salam and good-bye, talking softly with the patient etc. | The doctor showed respect to the patient perfectly. **The example of showing respect to the patient perfectly may be:** giving honor to an aged patient by standing up, helping an aged patient to sit down, giving Salam or at least replying when patients give Salam, talking softly with the patient etc. |
| **Examples of behavior showing disrespect might be:** Bargaining for money, using bad words, denying to provide treatment etc.; stopping the patient in the middle; talking in an authoritative tone, misbehaving, scolding etc.; getting the patient out of the room; “Do you know more than me? Then why did not you become a doctor?”-Telling such etc. | |  |  |

**History Taking**

32. **Listening to patient's complaints completely**

Patients expect that the doctors will start writing the prescription after listening to the symptoms in detail and completely. Assess the role of the doctor in this regard by 1 to 4 where 1-completely unsatisfactory and 4-completelysatisfactory. Possible observations are as follows:

| NA-------1-------2--------3--------4 | | | |
| --- | --- | --- | --- |
| The doctor did not listen to the patient attentively. The doctor stopped the patient while describing his complaint. | The doctor listened to some of the patient's complaints, but at some stage doctor stopped the patient either by verbal or non-verbal cues. Doctor had gone to the next step (examining, prescribing, etc.) before the patient finished describing his complaints. | The doctor listened to patient's complaints, did not stop the patient in the middle. But doctor had gone to the next step (examining, prescribing, etc.) before the patient finished describing his complaints. | The doctor heard the patient up to the end, did not interrupt (except necessary questions). He started next step only when the patient completed the description of the disease in details. |

33. **Listening to patient's complaints attentively**

Patients expect that the doctor will listen to them attentively with patience and that would be expressed by his behavior. Assess the role of the doctor in this regard by 1 to 4 where 1-completely unsatisfactory and 4-completelysatisfactory. Possible observations are as follows:

| NA-------1-------2--------3--------4 | | | |
| --- | --- | --- | --- |
| The doctor did not show attentiveness and patience in his behavior and language. | The doctor showed at least minimum attentiveness and patience in his behavior and language (e.g., At least one of the following things, **but did not ask questions**) | The doctor showed attentiveness and patience in his behavior and language to some extent (e.g., **Asking questions to learn more** and any two of the following). | The doctor showed attentiveness and patience clearly and fully by his behavior and language (all or most of the following). |
| **Behaviors indicating attentiveness and patience may be:** shaking head while talking, looking at the patient, asking questions to learn more, variation in tone, smiling face, some interest expressing words (e.g., Ok, hm etc.) etc. | | | |

34. **Counseling on social or family issues if related to the disease**

Patients expect that the doctor would help to solve the family problem that is related to his disease (e.g., torturing by husband, family feud, etc.) with the help of the concerned person of that area (e.g., political representative, administrative personnel, health sector personnel, etc.). Assess the role of the doctor in this regard by 1 to 4 where 1-completely unsatisfactory and 4-completelysatisfactory. Possible observations are as follows: **[Use N/A if there is no such patient.]**

| NA-------1-------2--------3--------4 | | | |
| --- | --- | --- | --- |
| The doctor did not take any step in this regard. | Though the doctor gave some suggestions in this matter, but did not get involved. | The doctor did not escape the matter rather tried to help the patient. However, it was not completely satisfactory. | The doctor informed the concerned person at once by calling or by some other means and made arrangement to solve the problem promptly. In this regard the role of the doctor was completely satisfactory. |

35. **Home visit by doctor**

Patients expect that doctor should visit their home for treatment when necessary. Assess the role of the doctor in this regard by 1 to 4 where 1-completely unsatisfactory and 4-completelysatisfactory. Possible observations are as follows: **[Use N/A if patients did not request doctor to visit his/her home.]**

| NA-------1-------2--------3--------4 | | | |
| --- | --- | --- | --- |
| He said ‘No’ rudely. | Did not go, however, explained it and even did not behave rudely. | Went reluctantly. | Went readily. |

**Examination**

36. **Examining the patient with care**

Patients expect that the doctor would do the necessary physical examination with care. Assess the role of the doctor in this regard by 1 to 4 where 1-completely unsatisfactory and 4-completelysatisfactory. Possible observations are as follows: **[Use ‘N/A’ if the patient does not come with a problem which require physical examination or if it is not clear whether the problem requires physical examination. If it is seemed that the problem requires physical examination, but doctor does not examine, fill in the field '1'.*Do not fill in 'N/A' field if the doctor conducted any physical examination.]***

| NA-------1-------2--------3--------4 | | | |
| --- | --- | --- | --- |
| The doctor did not examine the patient any more. | The doctor examined the patient at least once (only measured body temperature or pulse rate etc.). | The doctor measured the body temperature, blood pressure, pulse rate etc. (which were necessary) with some care. | The doctor measured the body temperature, blood pressure, pulse rate etc. and did all with care. |
| The example of examining the patient physically may be: telling the patient politely to fold up his sleeves, telling the patient that what he is going to do etc. | | | |

37. **Taking consent in particularly necessary conditions**

Patients expect that the doctor would take consent from the patient at least in some particularly necessary conditions. Assess the role of the doctor in this regard by 1 to 4 where 1-completely unsatisfactory and 4-completelysatisfactory. Possible observations are as follows: **[Use ‘N/A’ if there is no such particularly necessary case for taking consent (e.g., exposing the patient, touching, etc.) or if female patients do not come to a male doctor (e.g., male patient to male doctor, female patient to female doctor, and male patient to female doctor) or if it is not possible to observe somehow (e.g., if doctor or patient does not allow the observer in the room, if it falls under the exclusion criteria)]**

| NA-------1-------2--------3--------4 | | | |
| --- | --- | --- | --- |
| The doctor just ignored taking consent. | The doctor took consent at a minimum level. **As for example:** telling the female patient before doing any physical examination by a male doctor (e.g., I will do your ultra sonography, please fold up your sleeves as I am going to measure your blood pressure, etc.). | The doctor took consent somewhat properly. **As for example:** asking consent from the female patient in clear language before doing any physical examination by a male doctor (e.g., Mother, I will do this, do you agree?) | The doctor took consent perfectly. **As for example:** telling the patient before doing any examination and asking consent from the patient about it; keeping a female attendant if the male doctor examines a female patient, etc. |
| **The patients consider it *particularly necessary* to take consent when:** placing the stethoscope on the chest of a female patient by a male doctor; uncovering any covered part of the body or touching a part of the body while examining (except touching the forehead for fever). Though it is necessary to take consent in following cases, but it would not be possible to observe, as the observer is not allowed to stay in the room. The observer would go outside the room and use ‘NA’ in these situations. Examining the private parts of any patient; doing PR and PV; uncovering the abdomen of a female patient by a male doctor for examination; examining anybody by taking off the cloths; examining the appendix of a female patient by a male doctor etc. | | | |

38. **Taking consent in general**

A good practice is that, a doctor should inform the patient before doing any examination and take consent of the patient. Assess the role of the doctor in this regard by 1 to 4 where 1-completely unsatisfactory and 4-completelysatisfactory. Possible observations are as follows: **[Use ‘NA’ if it is not necessary to take consent (i.e., if no physical examination is done). *Do not use ‘NA’ if any physical examination is done.]***

| NA-------1-------2--------3--------4 | | | |
| --- | --- | --- | --- |
| The doctor examined in spite of the unwillingness and/or disagreement of the patient. | The doctor indirectly made the patient understand that he is going to do a physical examination. (E.g., uncovering the machine for measuring blood pressure, indicating the bed for doing examination etc.). As the patient did not say anything or did not protest, so it had been considered as 'implied consent'. | The doctor had **verbally** told the patient what he was going to do, but did not take his consent properly. **As for example**: I will examine your that organ (the name of the organ). | The doctor had said the patient that what he was going to do and took consent clearly. **As for example:** "Mother/father, I want to examine your that organ (name of the organ). Do you have any problem? Can I see it?" |

39. **Maintaining confidentiality of information**

Disclosure of information given by the patient about some sensitive issues may be harmful for him especially from the social point of view. Assess the doctor’s role for not disclosing the information outside by 1 to 4, where 1-completely unsatisfactory and 4-completely satisfactory. Possible observations are as follows: **[Use ‘NA’ if there were no such patient, if it could not be understood whether the information were sensitive, or if it was not possible to observe due to any reason (such as, if the doctor or patient does not allow the observer in the room, if it falls under the exclusion criteria).]**

| NA-------1-------2--------3--------4 | | | |
| --- | --- | --- | --- |
| The doctor did not play any role. | The doctor had minimal role. Some of the following points were observed. | The doctor had some role. Some of the following points were observed. | The doctor played role completely. Almost all of the following points were observed. |
| **Doctor can play the following roles:** Allowing nobody in the room except the doctor and the patient; if patient comes with his attendant, then talking to them separately; telling nothing if anybody wants to know anything about the disease or patient by introducing himself as the relative of the patient; assuring the patient that their conversation would be kept secret; ensuring that nobody heard their conversation from outside.  **Sensitive topics may include the following:** if an unmarried woman conceives, if a woman conceives though her husband lives abroad, tuberculosis, venereal disease, infertility. | | | |

**Prescription Writing**

40. **Suggestions on disease prevention and health promotion in general**

Patients expect that the doctor would not only treat the disease but also suggest some measures on disease prevention and health promotion. It may be or may not be directly related to the patient's disease. In this regard, assess the role of the doctor by 1 to 4, where 1-completely unsatisfactory and 4-completely satisfactory. Possible observations are as follows: **[This information is about common disease prevention and health promotion measures; specific prevention measures are given later]**

| NA-------1-------2--------3--------4 | | | |
| --- | --- | --- | --- |
| The doctor did not ask anything about the disease prevention and health promotion did not suggest anything in this regard. | Doctor indirectly told about some disease prevention and health promotion measures; but that came as part of the patient's disease condition. (The doctor told at least one of the following measures.) | The doctor asked the patient about the disease prevention and health promotion and also gave some suggestions in this regard. (The doctor told more than one of the following measures) | The doctor asked the patient about disease prevention and health promotion. He also advised the patient for leading a healthier life in details. |
| **Disease prevention and health promotion measures may be:** using sanitary latrines, habit of hand washing, vaccinating children, giving breast milk to the infants, physical activity or exercise, giving up smoking, general cleanliness, avoiding fatty foods, using germ-free water, eating nutritious food etc. | | | |

41. **Referral practice**

Patients expect that the doctor would refer the patient to another doctor immediately, if he cannot diagnose or treat the disease himself. In this regard, assess the role of the doctor by 1 to 4, where 1-completely unsatisfactory and 4-completely satisfactory. Possible observations are as follows: **[Use ‘NA’ if it is not necessary to refer the patient to another doctor or if it is not understood whether referring is required or not. If the patient required a referral (and the observer understood that), but if the patient was not referred, then fill out '1'. *Do not fill in the ‘NA’ if patient was referred to another doctor.]***

| NA-------1-------2--------3--------4 | | | |
| --- | --- | --- | --- |
| Even though the doctor knew that he would not be able to treat the disease, he did not refer the patient to another doctor. Or, the patient went away from the hospital at his own accord or was compelled to accept low-quality treatment. | The doctor did very late to realize that he would not be able to treat the patient, or he referred the patient only when the patient requested him. Or, he did not perform most of **the standard referral activities but one or two of the following:** explaining his limitations to the patient, telling where to take the patient, writing the address of the doctor/hospital where to take the patient, sending the information of the patient to the referred place beforehand etc. He referred the patient without care. | The doctor realized after a short time that he would not be able to treat the patient. He did all or some (at least 3) of **the following standard referral activities:** explaining his limitations to the patient, telling where to take the patient, writing the address of the doctor/hospital where to take the patient, sending the information of the patient to the referred place beforehand etc. | The doctor realized quickly that he would not be able to treat the patient, explained his limitations to the patient, told where to take the patient, wrote the address of the doctor/hospital where to take the patient, sent the information of the patient to the referred place beforehand etc. He satisfactorily did all the tasks related with referring the patient. |
| **Note:** If the patient is sent for a diagnostic test, or if he is advised to consult the doctor with the result of the test- then it is not considered as a referral. | | | |

42. **Consultation with colleagues if in confusion**

Patients expect that the doctor would discuss with his colleague, another nearest doctor or anybody having knowledge about the disease if the doctor has some confusion or does not understand anything clearly about the treatment. In this regard, assess the role of the doctor by 1 to 4, where 1-completely unsatisfactory and 4-completely satisfactory. Possible observations are as follows: **[Fill in the ‘NA’ field if the doctor is not unable to provide treatment, or if it is not understood whether he is unable to provide treatment. If the doctor’s inability is visible to the observer, but if the doctor does not consult with anybody, fill out '1'. *If the doctor consulted with others, do not fill out 'NA'.* ]**

| NA-------1-------2--------3--------4 | | | |
| --- | --- | --- | --- |
| Although the inability of the doctor for treating the disease was observed (such as inability to understand X-ray, ECG, ultra sonogram, inability to diagnose skin diseases confidently etc.), he did not ask anybody (nearest doctor, SACMO, nurse or by phone call). | Although the inability of the doctor for treating the disease was observed (such as inability to understand X-ray, ECG and ultra sonogram, inability to diagnose skin diseases confidently etc.), he did not tell it directly to his patient. He asked somebody (nearest doctor, SACMO, nurse or by phone call) with hesitation and unwillingness. | Although the inability of the doctor for treating the disease was observed (such as inability to understand X-ray, ECG and ultra sonogram, inability to diagnose skin diseases confidently etc.), he did not tell it directly to his patient. He asked somebody (nearest doctor, SACMO, nurse or by phone call) about it promptly. | The doctor clarified his inability or confusion to the patient and provided treatment after discussing with a related person promptly. |

43. **Allowing patients to choose doctors**

Often patients cannot choose the right doctor (Neurologist, Cardiologist etc.) for their diseases. Often they want to go to male/female doctor and expect to consult with the nearest doctor. They expect that they would consult with the doctor and the doctor would provide suggestion free of cost. In this regard, assess the role of the doctor by 1 to 4, where 1-completely unsatisfactory and 4-completely satisfactory. Possible observations are as follows: **[Fill in the ‘NA’ field if no patient comes with such issue.]**

| NA-------1-------2--------3--------4 | | | |
| --- | --- | --- | --- |
| The doctor did not suggest anything when patient came with such purpose. | The doctor gave suggestion but rudely or it was not suitable for the patient. | The doctor was reluctant to suggest. However, his behavior was not rude. | The doctor suggested the patient sincerely and cordially. |

44. **Giving courage and reassurance**

Patient wants courage and assurance from the doctor. In this regard, assess the role of the doctor by 1 to 4, where 1-completely unsatisfactory and 4-completely satisfactory. Possible observations are as follows:

| NA-------1-------2--------3--------4 | | | |
| --- | --- | --- | --- |
| The doctor neither said anything nor behaved in such a way that expressed reassurance. | The doctor reassured either verbally or nonverbally (speech or behavior). | The doctor reassured both verbally and nonverbally. (There should be both speech and behavior). | The doctor showed most of the reassurance expressing speech or behavior (there should be both speech and behavior). |
| **Reassurance expressing speech and behavior may be: You have no problem;you will be all right; nothing has happened to you; there is nothing to be worried; I would be able to cure your disease, inshallah, etc.**-such type of speech; putting hands on the shoulder of the patient, giving him courage by holding his hand, giving courage by putting hand on the body-such type of behavior | | | |

45. **Earning trust of patients**

Patients want to have trust on the doctor. The doctor should not tell or do anything, which might breach the trust; rather he should behave for earning trust. Here ‘trust’ means **“The doctor advised for maximizing the patient's benefit, not for maximizing his own benefit."** In this regard, assess the role of the doctor by 1 to 4, where 1-completely unsatisfactory and 4-completely satisfactory. Possible observations are as follows:

| NA-------1-------2--------3--------4 | | | |
| --- | --- | --- | --- |
| Most of the behaviors (at least two) of the doctor were such that might break the trust of the patient. | Some behaviors (at least one) of the doctor were trust breaking. | The doctor did not behave such that might cause breach of trust of the patient. However, he also did not do anything for earning the trust of the patient. | The doctor did not behave such that might cause breach of trust of the patient. However, he tried to earn the trust of the patient. |
| **Examples of such behavior that may cause breach of trust:** telling the patient to do test from any specific diagnostic center (but if the patient himself asks where to do the test and the doctor tells the name in response, then it will not be considered as breach of trust), encouraging to buy medicines of a specific pharmaceutical company, telling the patient under consultation of a govt. doctor to go to a private clinic, seeing private patients by a public doctor during office hour (moonlighting) etc.  **Examples of activities to gain the trust:** Explaining the necessity to the patient if any test has been given. | | | |

46. **Service oriented, not businesslike behavior**

Patients expect service-oriented behavior from the doctor and consider business-oriented behavior as unwanted. In this regard, assess the role of the doctor by 1 to 4, where 1-completely unsatisfactory and 4-completely satisfactory. Possible observations are as follows:

| NA-------1-------2--------3--------4 | | | |
| --- | --- | --- | --- |
| Most of the behaviors (at least two) of the doctor were such that the patient might think those as business oriented behavior. | Some behaviors (at least one) of the doctor might be considered as of business oriented to the patient. | The doctor did not do anything that might be considered as business oriented to the patient. However, the doctor’s behavior was not service oriented also. | The doctor did not do anything that might be considered as business oriented to the patient. Rather, the doctor’s behavior was service oriented. |
| **Behaviors expressing business oriented behavior may be:** telling the patient to do test from any specific diagnostic center, encouraging to buy medicines of a specific pharmaceutical company, taking money from patients forcibly, telling the patient under consultation of a govt. doctor to go to a private clinic, etc.  **Examples of service-oriented behaviors may be:** asking the patient’s ability to bear the cost of treatment, if necessary assisting the patient in getting low-cost medical care and so on. | | | |

47. **Not sending patients to specific diagnostic centers**

Patients most dislike if the doctor tells the patient to do diagnostic tests from any specific diagnostic center. In this regard, assess the role of the doctor by 1 to 4, where 1-completely unsatisfactory and 4-completely satisfactory. Possible observations are as follows: **[Fill in the ‘NA’ field if no test is prescribed.]**

| NA-------1-------2--------3--------4 | | | |
| --- | --- | --- | --- |
| The doctor forced the patient to do tests from a specific diagnostic center. | The doctor advised the patient to do tests from a specific diagnostic center but did not force him to do so. | The doctor did not advise the patient directly to do tests from a specific diagnostic center. However, he indirectly did (such as, do you want to do test from any good diagnostic center?). | The doctor did not suggest any specific place for doing tests; even he did not do so indirectly. |
| **Note:** It would not be considered as negative if the doctor suggests doing tests from the upazila health complex or any other public organizations; because it is possible at low prices and the doctor does not have the chance of any benefit. | | | |

48. **Involving patients in care related decision making**

A good practice is that, the patient should participate in making decisions regarding treatment. He should be informed about different treatment options, treatment cost and advantages and disadvantages of each option. The doctor should help the patient to choose the best option by considering patient's personal and social conditions and the doctor should respect the patient’s choice. In this regard, assess the role of the doctor by 1 to 4, where 1-completely unsatisfactory and 4-completely satisfactory. Possible observations are as follows:

| NA-------1-------2--------3--------4 | | | |
| --- | --- | --- | --- |
| The doctor did not make the patient participative in making decisions regarding treatment. | The doctor told the patient about different treatment options. He informed about advantages and disadvantages of those options. | The doctor told the patient about different treatment options, informed about advantages and disadvantages of those options and helped the patient to choose the best option for him by considering patient's personal and social conditions. | The doctor told the patient about different treatment options. He gave idea about treatment cost and informed about advantages and disadvantages of those options. He helped the patient to choose the best option for him by considering patient's personal and social conditions. The doctor honored the patient’s choice. |

49. **Considering individual need of the patient**

Each patient is an individual person and the patient’s environment (context) is also different; so their needs are also different. Patients expect that the doctor would provide treatment to comply with the patient's individual need. In this regard, assess the role of the doctor by 1 to 4, where 1-completely unsatisfactory and 4-completely satisfactory. Possible observations are as follows: **[Fill in the ‘NA' field if patient does not inform the doctor about his problem to comply with the prescribed treatment.]**

| NA-------1-------2--------3--------4 | | | |
| --- | --- | --- | --- |
| The patient informed the doctor about his problem to receive the prescribed treatment. The doctor did not adjust his treatment; rather rudely informed that the issue was not under his jurisdiction or that he is unable to solve the matter. | The patient informed the doctor about his problem to receive the prescribed treatment. The doctor did not adjust his treatment; he politely informed that the issue is not under his jurisdiction or that he is unable to solve the matter. | The patient informed the doctor about his problem to receive the prescribed treatment. Then the doctor adjusted his treatment. However, the doctor did not make sure whether the patient would be able to follow the changed prescription. | The patient informed the doctor about his problem to receive the prescribed treatment. The doctor adjusted his treatment after listening to the patient attentively. The doctor made sure that the patient would be able to follow the changed prescription. |
| **Examples of contextual and the individual need of the patient may be:** considering the obstacles of patient's personal and social life (referring the patient to a distant place, coordinating the treatment schedule with children's examination and the patient's job, etc.). | | | |

50. **Considering religious and cultural orientation of the patient**

Doctors should prescribe treatment considering the religious and cultural orientation of the patient. What was the status of such cultural sensitivity at the suggestion of the doctor? In this regard, assess the role of the doctor by 1 to 4, where 1-completely unsatisfactory and 4-completely satisfactory. Possible observations are as follows:

| NA-------1-------2--------3--------4 | | | |
| --- | --- | --- | --- |
| The doctor was culturally insensitive. | The doctor did not do any kind of culturally insensitive behavior. However, he also did not leave an example of a culturally sensitive behavior. | The doctor demonstrated at least minimum level of cultural sensitivity during consultation. | The doctor demonstrated cultural sensitivity clearly more than once in various steps of consultation. |
| **Examples of cultural sensitivity may be:** making adjustment while giving medicine to a Muslim patient during Ramadan, giving advice to the patient to eat less fried food at ‘iftari’, telling the patient to eat fruits that are available during that season (or refraining from suggesting out of season fruits), not giving advice of doing or eating anything which is religiously prohibited, giving idea about the disease and treatment by using some local languages (e.g., using ‘giving gas’ instead of nebulization, ‘Jor chumka’ instead of febrile convulsion, ‘lukewarm water’ for giving idea of hot water etc.), using simple examples to explain the advice given (such as RC Cola Bottle head equivalent drugs, mixing a pinch of salt, one handful gur etc.), explaining in plain Bangla language after using medical terminology (e.g., *puj* instead of 'pus cell', *hojom hoe jabe* instead of 'it will be absorbed', etc.) and so on.  **Examples of cultural insensitivity may be:** Suggesting any diet to the patient which is religiously prohibited, advising such fruit or food which is not available in that season; using medical terminology; using very formal language which the patient cannot understand; wearing such a dress which is socio-culturally unacceptable. | | | |

51. **Facilitating utilization of local resources**

It is expected from the doctor that they would facilitate the service at the locality of the patient. So, doctors should know about the resources available in the locality of the patient and give suggestion accordingly. In this regard, assess the role of the doctor by 1 to 4, where 1-completely unsatisfactory and 4-completely satisfactory. Possible observations are as follows: **[Fill in the ‘NA' field if no such patient comes or if it cannot be understood.]**

| NA-------1-------2--------3--------4 | | | |
| --- | --- | --- | --- |
| The doctor did not want to know about the local resources; even he did not take it seriously when patient told. | The doctor did not want to know about local resource on his own. However, he adjusted his treatment when the patient told. | The doctor wanted to know about the local resource on his own, he adjusted his treatment depending on the patient's answers. | The doctor wanted to know about the local resource on his own, took the patient’s answers seriously, adjusted his treatment depending on the patient's answers and gave necessary advices. |
| **Examples of local resource maybe:** there may be a service provider in the patient’s community who may inject medicine or saline; The patient may have a relative at home who can help the patient in the treatment (reminding medicine, exercise etc.). | | | |

52. **Considering socio-economic status of the patient**

Patients expect that the doctors would consider the financial strength of the patients and help the patients to get treatment within their ability. In this regard, assess the role of the doctor by 1 to 4, where 1-completely unsatisfactory and 4-completely satisfactory. Possible observations are as follows: **[Fill in the ‘Not Applicable’ field if all treatment is provided at free of cost or if the treatment is very cheap (Diarrhea, common cold and fever etc.).]**

| NA-------1-------2--------3--------4 | | | |
| --- | --- | --- | --- |
| None of the three steps of financial assistance to the patient was followed. The doctor even did not help the patient when needed; he did not explain the patient and even he did not show sympathy. | One of the three steps of financial assistance to the patient (examples are given below) was followed. Even he helped the patient when needed (at least one of the following helps); the doctor explained when he could not help and showed sympathy. | Two among three steps of financial assistance to the patient (examples are given below) were partially followed. Even he helped the patient when needed (at least one of the following helps); the doctor explained when he could not help and showed sympathy. | Two among three steps of financial assistance to the patient (examples are given below) were fully followed. Even he helped the patient when needed (more than one of the following helps); the doctor explained when he could not help and showed sympathy. |
| **It is necessary to follow three steps for providing financial assistance to the patient:** Trying to understand the financial condition of the patient; giving idea about treatment cost; helping the patient if necessary.  **Example of trying to understand the financial condition of the patient may be:** Asking the patient directly about his income or whether he would be able to bear the treatment cost; Asking him indirectly (such as, asking his profession); if the patient tells. Beside these, it might be guessed by observing the patient’s conversation, behavior and clothing or the doctor might have idea about local people-however, but it is difficult to understand through observation.  **Example of giving idea about cost of treatment may be:** How much would be needed to complete the treatment; how long the treatment may continue; what impact the patient would be able to put on his ability of income during and after receiving treatment.  **Example of helping the poor patient may be:** Prescribing low cost antibiotics; taking less or no consultation fee (in case of private doctors); helping patients from ‘poor fund’; helping forgetting free medicines from the hospital (in case of government doctors);giving time and advice to collect money; focusing on the history and physical examination to avoid investigation; prescribing the essential tests only; cutting the commission paid to the doctor for each test; recommending that treatment method to the patient which saves money (meeting the nutritional needs from domestic sources, suggesting the pregnant woman to spend money for nutritious food instead of repeated ultra sonography etc.) and so on. | | | |

53. **Trying to understand socio-economic status of the patient**

Patients expect that the doctor would try to understand the socio-economic condition of the patient before providing treatment. In this regard, assess the role of the doctor by 1 to 4, where 1-completely unsatisfactory and 4-completely satisfactory. Possible observations are as follows: **[Fill in the ‘Not Applicable’ field if the patient is visibly financially solvent or if treatment is given at free of cost or if treatment cost of the disease is smaller amount (diarrhea, common cold and fever etc.).]**

| NA-------1-------2--------3--------4 | | | |
| --- | --- | --- | --- |
| The doctor did not try to understand the patient's financial condition. | The doctor heard and understood when the patient told willingly. | The doctor indirectly asked him (such as, he asked the profession of the patient) that whether he would be able to bear the cost of the treatment. | The doctor directly asked him that whether he would be able to bear the cost of the treatment. |

54. **Informing the cost of treatment/ financial counseling**

Patients expect that the doctors would give them idea about treatment cost before starting treatment. In this regard, assess the role of the doctor by 1 to 4, where 1-completely unsatisfactory and 4-completely satisfactory. Possible observations are as follows: **[If all medications are given free of cost, or if the treatment is very cheap (diarrhea, common cold) then fill in the ‘NA 'field]**

| NA-------1-------2--------3--------4 | | | |
| --- | --- | --- | --- |
| The doctor did not give any idea about the cost of treatment to the patient, even if the patient asked. | The doctor gave minimum idea to the patient about the treatment cost and did so when patient wanted to know. **Examples might be:** how much will it cost to complete the treatment. | The doctor gave rough idea to the patient about the treatment cost or did so when the patient asked. **In this case examples of giving idea might be**: how much will it cost to complete the treatment, how long treatment may continue on etc. | The doctor himself told the patient in details about the treatment cost. **Examples of giving idea might be:** how much will it cost to complete the treatment, how long treatment may continue on and what would be the impact on the earning ability of the patient after completion of the treatment etc. |

55. **Providing financial assistance if needed**

Patients expect that the doctor would help them if they become unable to bear the cost of the treatment. In this regard, assess the role of the doctor by 1 to 4, where 1-completely unsatisfactory and 4-completely satisfactory. Possible observations are as follows: **[If the patient is visibly affluent, or the doctor confirmed his ability to bear the cost by asking the patient, then fill in the ‘NA' field.]**

| NA-------1-------2--------3--------4 | | | |
| --- | --- | --- | --- |
| The doctor did not help the patient by any means. | The doctor did minimum helps from the following list (at least one) to the patient. | The doctor did some helps from the following list (at least two) to the patient. | The doctor did almost all helps from following list to the patient. |
| **The examples of helping the poor patient may be:** Prescribing low cost antibiotics, taking less or no consultation fee (in case of private doctors), providing financial assistance to the poor patients, helping in getting free medicines from the hospital (in case of government doctors), giving time and advice to obtain money for treatment, trying to focus on the history and physical examination to avoid investigation, prescribing the essential tests only, deducting the commission paid to the doctor for each test, recommending the treatment method that saves money (to meet the nutritional needs from domestic sources, suggesting the pregnant woman to spend money for nutritious food instead of repeated ultra sonography etc.) and so on. | | | |

56. **Facilitating follow-up**

Patients expect that the doctors would facilitate post treatment follow-up and give them a follow-up plan. In this regard, assess the role of the doctor by 1 to 4, where 1-completely unsatisfactory and 4-completely satisfactory. Possible observations are as follows:

| NA-------1-------2--------3--------4 | | | |
| --- | --- | --- | --- |
| The doctor did not give any follow-up plan to the patient. | The doctor gave **minimum** follow-up plan to the patient. **At least** he told when to come to the doctor again and what would be the cost of follow-up. | The doctor gave the patient a somewhat fair follow-up plan. At least he told when to come to the doctor again and what would be the cost of follow-up. Beside this, at least one of the following points was included. | The doctor willingly gave the patient a complete follow-up plan. Almost all of the following points were included. |
| **Complete follow-up plan could be:** When the patient would meet the doctor again; in which case the patient should contact the doctor before; if necessary, how the patient can reach the doctor; providing mobile number to the patient; telling about follow-up costs; follow-up should be at free of cost; to write down what the patient should come up with at the time of follow-up (or at least tell); telling to inform the doctor immediately if any of the side effects of treatment arise etc.  **Note:** Often doctors suggest the patients to meet them again after doing any test. It would not be regarded as follow-up because it is not a part of sensitivity but a part of the treatment process. | | | |

**Explanations and Questions**

57. **Quantity of issues explained and the quality of explanation**

Patients expect that the doctor would explain everything to them, such as cause of the disease, diagnosis (at least the name of the disease), prognosis and severity, treatment (at least explaining the prescription), side effects of the medicines (if any), report of diagnostic tests (if any), preventive measures of disease (Diet) etc.; he would do it by himself (that means he would not give this responsibility to his assistant or pharmacist, rather he would tell it) and ask the patient whether he has understood. In this regard, assess the role of the doctor by 1 to 4, where 1-completely unsatisfactory and 4-completely satisfactory. Possible observations are as follows: **[Follow two steps for answering this question: at first, guess a score on the basis of three parameters of giving explanation. Later, adjust the score on the basis of the number and quality of explained issues.]**

| NA-------1-------2--------3--------4 | | | |
| --- | --- | --- | --- |
| Explained, he himself explained, asked whether understood – no one of these three points is positive. | Explained, he himself explained, asked whether understood –one of these three points is positive. | Explained, he himself explained, asked whether understood-two of these three issues are positive. | Explained, he himself explained, asked whether understood- all of these three issues are positive. |

58. **Quantity of issues explained**

Patients expect the doctor would explain everything to them, such as cause of the disease, diagnosis (at least the name of the disease), prognosis and severity, treatment (at least explaining prescription), the side effects of the drugs (if any), the result of diagnostic tests (if any), preventive measures of disease (Diet) etc. In this regard, assess the role of the doctor by 1 to 4, where 1-completely unsatisfactory and 4-completely satisfactory. Possible observations are as follows:

| NA-------1-------2--------3--------4 | | | |
| --- | --- | --- | --- |
| The doctor did not explain anything to the patient. He only wrote the prescription. | The doctor explained at least one thing to the patient. Such as: diagnosis (at least the name of the disease), treatment (at least explaining prescription) etc. | The doctor explained many issues to the patient. Such as: diagnosis (at least the name of the disease), treatment (at least explaining prescription), the result of diagnostic tests (if any), preventive measures of disease (Diet) etc. | The doctor explained everything to the patient. Such as: cause of the disease, diagnosis (at least the name of the disease), treatment (at least explaining prescription), the side effects of the drugs (if any), the result of diagnostic tests (if any), preventive measures of disease (Diet) etc. |

59. **Explaining everything to the patient by the doctor himself**

Patients expect that the doctor (i.e., not her/his assistant, pharmacist or anyone else) would explain different aspects about the patient’s disease (in this case, diagnosis, treatment and diet) to them. S/he would not leave this task on anybody else (such as, his assistant, pharmacist, etc.). In this regard, assess the role of the doctor by 1 to 4, where 1-completely unsatisfactory and 4-completely satisfactory. Possible observations are as follows: [**Fill in the ‘Not Applicable’ field if at least three issues (such as diagnosis, treatment and diet) are not explained.]**

| NA-------1-------2--------3--------4 | | | |
| --- | --- | --- | --- |
| The doctor told the patient to go to another person (assistant, pharmacist, any other person) for explanation of all three issues. | The doctor explained one of the three issues; he told the patient to go to another person (assistant, pharmacist, any other person) for explanation of two issues. | The doctor explained two of the three issues; he told the patient to go to another person (assistant, pharmacist, any other person) for explanation of one issue. | The doctor explained diagnosis of the disease (name of the disease), treatment and diet to the patient. He did not involve any one else for this task. |

60. **Asking patient if s/he understood the explanation**

It is extremely important that the patient understands all suggestions or explanations given by the doctor. The doctor should be sure that the patient understands him. In this regard, assess the role of the doctor by 1 to 4, where 1-completely unsatisfactory and 4-completely satisfactory. Possible observations are as follows: **[If the doctor does not explain anything, then fill in the ‘Not Applicable’ field; because there is no question of understanding, if he does not explain anything.]**

| NA-------1-------2--------3--------4 | | | |
| --- | --- | --- | --- |
| The doctor did not ask the patient about his understanding after explaining him the cause of the disease, diagnosis (name of the disease), prognosis, treatment etc. | The doctor asked the patient about his understanding after explaining him at least one (especially name of the disease and/or treatment) of the following issues, such as, cause of the disease, diagnosis (name of the disease), prognosis, treatment etc. | The doctor asked the patient about his understanding after explaining him at least two or more of the following issues, such as, cause of the disease, diagnosis (name of the disease), prognosis, treatment etc. | The doctor asked the patient about his understanding after explaining him each of the following issues, such as, cause of the disease, diagnosis (name of the disease), prognosis, treatment etc. |

61. **Explaining the cause of disease to the patient**

Patients expect that the doctor would explain the cause of the disease like why the disease occurred, what may be the causes of the disease etc., he would do it on his own (that means he would not give responsibility to his assistant, pharmacist, rather he would tell it) and would ask whether the patient has understood. In this regard, assess the role of the doctor by 1 to 4, where 1-completely unsatisfactory and 4-completely satisfactory. Possible observations are as follows: **[If no patient comes with such diseases like injury, common cold and fever etc. where it is necessary to tell the causes, fill in the ‘Not Applicable’ field.][Follow two steps for answering this question: at first, guess a score on the basis of three parameters of giving explanation. Later, adjust the score on the basis of quality of explained issues.]**

| NA-------1-------2--------3--------4 | | | |
| --- | --- | --- | --- |
| Explained the cause of the disease, he himself explained, asked whether understood – none of these three points is positive. | Explained the cause of the disease, he himself explained, asked whether understood –one of these three points is positive. | Explained the cause of the disease, he himself explained, asked whether understood- two of these three issues are positive. | Explained the cause of the disease, he himself explained, asked whether understood- all of these three issues are positive. |

62. **Explaining the diagnosis of disease to the patient**

Patients expect that the doctor would explain in details about the diagnosis (that means the name of the disease) of their diseases (However, it should be told in such a way that it does not create panic). In this regard, assess the role of the doctor by 1 to 4, where 1-completely unsatisfactory and 4-completely satisfactory. Possible observations are as follows: **[If no patient comes with such diseases like injury, common cold and fever etc. where it is necessary to tell about the diagnosis, fill in the ‘Not Applicable’ field.][Follow two steps for answering this question: at first, guess a score on the basis of three parameters of giving explanation. Later, adjust the score on the basis of quality of explained issues.]**

| NA-------1-------2--------3--------4 | | | |
| --- | --- | --- | --- |
| Explained about diagnosis, he himself explained, asked whether understood – none of these three points is positive. | Explained about diagnosis, he himself explained, asked whether understood –one of these three points is positive. | Explained about diagnosis, he himself explained, asked whether understood- two of these three issues are positive. | Explained about diagnosis, he himself explained, asked whether understood- all of these three issues are positive. |

63. **Explaining the prognosis of disease to the patient**

Patients expect that the doctor would explain the severity of the disease, prognosis (recovery, consequence etc.) etc. in details. In this regard, assess the role of the doctor by 1 to 4, where 1-completely unsatisfactory and 4-completely satisfactory. Possible observations are as follows: **[If no patient comes with such diseases like common cold and fever etc. where it is necessary to tell about the prognosis, fill in the ‘Not Applicable’ field.][Follow two steps for answering this question: at first, guess a score on the basis of three parameters of giving explanation. Later, adjust the score on the basis of quality of explained issues.]**

| NA-------1-------2--------3--------4 | | | |
| --- | --- | --- | --- |
| Explained about severity and prognosis, he himself explained, asked whether understood – none of these three points is positive. | Explained about severity and prognosis, he himself explained, asked whether understood –one of these three points is positive. | Explained about severity and prognosis, he himself explained, asked whether understood- two of these three issues are positive. | Explained about severity and prognosis, he himself explained, asked whether understood- all of these three issues are positive. |

64. **Explaining the treatment to the patient**

Patients expect that the doctor would explain about the treatment of their diseases like which medicines have been given and why, how to take those medicines etc. In this regard, assess the role of the doctor by 1 to 4, where 1-completely unsatisfactory and 4-completely satisfactory. Possible observations are as follows: **[Fill in the ‘Not applicable’ field if no treatment is given (such as, if patient is referred or admitted in the hospital).] [Follow two steps for answering this question: at first, guess a score on the basis of three parameters of giving explanation. Later, adjust the score on the basis of quality of explained issues.]**

| NA-------1-------2--------3--------4 | | | |
| --- | --- | --- | --- |
| Explained about treatment, he himself explained, asked whether understood – none of these three points is positive. | Explained about treatment, he himself explained, asked whether understood –one of these three points is positive. | Explained about treatment, he himself explained, asked whether understood- two of these three issues are positive. | Explained about treatment, he himself explained, asked whether understood- all of these three issues are positive. |

65. **Explaining the preventive aspects to the patient**

Patient expects that the doctor along with the treatment of the disease would also explain in details about diet, which foods are allowed and which are forbidden, prevention of the disease for which he has gone to the doctor, how to remain away from it etc. as well as lifestyle modification, preventive advice etc. In this regard, assess the role of the doctor by 1 to 4, where 1-completely unsatisfactory and 4-completely satisfactory. Possible observations are as follows: **[If no patient comes with such issues or if it is not clear whether it is necessary to tell such things, fill in the ‘Not Applicable’ field.][Follow two steps for answering this question: at first, guess a score on the basis of three parameters of giving explanation. Later, adjust the score on the basis of quality of explained issues.]**

| NA-------1-------2--------3--------4 | | | |
| --- | --- | --- | --- |
| Explained about diet, he himself explained, asked whether understood – none of these three points is positive. | Explained about diet, he himself explained, asked whether understood –one of these three points is positive. | Explained about diet, he himself explained, asked whether understood- two of these three issues are positive. | Explained about diet, he himself explained, asked whether understood- all of these three issues are positive. |
| **Examples of lifestyle modification related advices may be:** In case of diarrheal patients, how diarrhea spreads and how to escape from the diarrhea (hand washing, use of sanitary latrines, etc.); protecting the child from catching cold who is suffering from pneumonia; advising the patient suffering from venereal diseases to use condom; maintaining cleanliness and drinking more water in case of UTI; eating less spicy food, drinking more water in case of PUD's; avoiding sweet foods in case of Diabetic patients; avoiding oily food, weight loss, eating less, taking precautions for preventing common fever and cold (wearing warm clothes, drinking warm water) in case of cardiac diseases and so on. | | | |

66. **Explaining the side effects of the treatment to the patient**

Medicines, which are given for some diseases, have side effects such as medicine for tuberculosis. Patients expect that the doctor would warn them if there is any such side effect. However, it should be done in such a way that it does not create unnecessary fear about the medicine. In this regard, assess the role of the doctor by 1 to 4, where 1-completely unsatisfactory and 4-completely satisfactory. Possible observations are as follows: **[If no patient comes with such issues or if it is not understandable to the observer, fill in the ‘Not Applicable’ field.][Follow two steps for answering this question: at first, guess a score on the basis of three parameters of giving explanation. Later, adjust the score on the basis of quality of explained issues.]**

| NA-------1-------2--------3--------4 | | | |
| --- | --- | --- | --- |
| Explained about side effects, he himself explained, asked whether understood – none of these three points is positive. | Explained about side effects, he himself explained, asked whether understood –one of these three points is positive. | Explained about side effects, he himself explained, asked whether understood- two of these three issues are positive. | Explained about side effects, he himself explained, asked whether understood- all of these three issues are positive. |

67. **Explaining the result of tests to the patient**

If the patient does any diagnostic test (during any previous visit, at another doctor's advice or on his own) and shows the report to the doctor during consultation, the doctor should explain it to the patient. In this regard, assess the role of the doctor by 1 to 4, where 1-completely unsatisfactory and 4-completely satisfactory. Possible observations are as follows: **[If no patient comes with such issues, then fill in the ‘Not Applicable’ field.][Follow two steps for answering this question: at first, guess a score on the basis of three parameters of giving explanation. Later, adjust the score on the basis of quality of explained issues.]**

| NA-------1-------2--------3--------4 | | | |
| --- | --- | --- | --- |
| Explained the test report, he himself explained, asked whether understood – none of these three points is positive. | Explained the test report, he himself explained, asked whether understood –one of these three points is positive. | Explained the test report, he himself explained, asked whether understood- two of these three issues are positive. | Explained the test report, he himself explained, asked whether understood- all of these three issues are positive. |

68. **Allowing patient to ask questions**

Patients expect that the doctor would give them the opportunity to ask questions and also give appropriate answers to the patients. In this regard, assess the role of the doctor by 1 to 4, where 1-completely unsatisfactory and 4-completely satisfactory. Possible observations are as follows: **[If the patient did not ask any question fill in the 'NA' field.]**

| NA-------1-------2--------3--------4 | | | |
| --- | --- | --- | --- |
| The doctor did not answer the patient’s questions or snubbed him. | The doctor himself did not ask if the patient has any question. However, he briefly answered some questions of the patient (example: replied in one sentence, such as, when the patient asked about his disease, he said: skin disease, infection, etc.). If patient asked irrelevant questions, he was slightly resent. | The doctor himself did not ask if the patient has any question. However, he answered almost all the questions of the patient in somewhat details and accurately. Even when the patient asked irrelevant questions, he did not misbehave, but did not explain it. | The doctor himself asked the patient if he has any questions. He answered all the questions of the patient in details and appropriate way. Even when the patient asked irrelevant questions, he did not misbehave, rather he explained the patient that the question is not relevant. |
| **The patients usually have questions about:** why he is not getting well even after taking medicine for a long time; what is the name of his disease; treatment related any other question; when he would come to the doctor next time; what kind of food to eat.  **The questions of the patients that might be considered irrelevant:** asking the same question repeatedly; questions about price of medicines are considered irrelevant to the doctors; answering the same questions again and again to various person who came with the patient; questions which are not related with the disease, to refrain from any suggestions (such as smoking, sexual intercourse, etc.); asking guarantee about patient’s recovery etc. | | | |

69. **Answering patient's questions by doctor her/himself**

Patients expect that the doctor would reply if the patient has any question (in this case, at least three questions). S/he would not leave this task on anybody else (e.g., her/his assistant, pharmacist etc.). In this regard, assess the role of the doctor by 1 to 4, where 1-completely unsatisfactory and 4-completely satisfactory. Possible observations are as follows: **[If patient does not ask at least three questions, fill in the ‘Not Applicable’ field.]**

| NA-------1-------2--------3--------4 | | | |
| --- | --- | --- | --- |
| The doctor did not answer any of the three questions; he told the patient to go to another person for each question (assistant, pharmacist, any other person). | The doctor replied at least one question. However, he told the patient to go to another person for answer of the two questions (assistant, pharmacist, any other person). | The doctor answered at least two questions. However, he told the patient to go to another person for the answer of one question (assistant, pharmacist, any other person). | The doctor answered all three questions of the patient. |

70. **Keeping patience in patient's irrelevant questions**

Many of our patients often ask irrelevant questions, as they do not know what to ask about the disease. The patients expect that the doctor would keep patience about their questions and tell them nicely. In this regard, assess the role of the doctor by 1 to 4, where 1-completely unsatisfactory and 4-completely satisfactory. Possible observations are as follows: **[If the patient does not have any questions or if there is not an irrelevant question, fill in the ‘Not Applicable’ field.]**

| NA-------1-------2--------3--------4 | | | |
| --- | --- | --- | --- |
| When the patient asked irrelevant question, the doctor insulted him. He did not explain that the question is irrelevant to his disease. He might be angry with him. | When the patient asked irrelevant question, the doctor told him that the question is irrelevant to his disease. He did not become angry with him, but his behavior expressed annoyance. He did not say what might be the relevant questions. | When the patient asked any irrelevant question, the doctor did not insult him, rather smiled and explained nicely that the question is irrelevant to his disease. He neither become angry with him nor insulted him. However he did not say what might be the relevant questions. | When the patient asked any irrelevant question, the doctor did not insult him, rather smiled and explained nicely that the question is irrelevant to his disease. He neither become angry with him nor insulted him. He told what might be the relevant question and also answered it. |
| **The questions of the patients that might be considered irrelevant:** asking the same question repeatedly; questions about price of medicines are considered irrelevant to the doctors; answering the same questions again and again to various person who came with the patient; questions which are not related with the disease, to refrain from any suggestions (such as smoking, sexual intercourse, etc.); asking guarantee about patient’s recovery etc. | | | |

71. **Encouraging patient to ask questions**

Some of the behavior of the doctor discourages patients to ask questions. Patients expect that they would not be discouraged by any behavior of the doctor; rather the doctor would behave such that would provide courage and encourage them to ask questions. In this regard, assess the role of the doctor by 1 to 4, where 1-completely unsatisfactory and 4-completely satisfactory. Possible observations are as follows:

| NA-------1-------2--------3--------4 | | | |
| --- | --- | --- | --- |
| The patients were greatly discouraged to ask questions by the words or behavior of the doctor. **Examples of discouraging behavior may be:** repeatedly looking at the clock, giving reminder to the patient to be short, writing prescription while answering the question, ultra-seriousness, answering very shortly (in one word), answering in nagging tone, remaining many patients together at the queue, "you understand more," or "you are the doctor" - telling such neglectful words etc. | The patients might be slightly discouraged to ask questions by the words or behavior of the doctor. **Examples of such behavior could be:** repeatedly looking at the clock, writing prescription while answering the question, answering very shortly (in one word), waiting many patients at the queue, etc. | The words or behavior of the doctor did not discourage patients to ask question. However, the doctor also did not behave in such way to provide courage or to encourage the patients for asking questions. **Example of encouraging conduct may be:** smile answered, carefully listening to the questions, etc. | The words or behavior of the doctor did not discourage patients to ask question; rather the doctor behaved in such way that provided courage or encouraged the patients for asking questions. **Example of encouraging conduct may be:** smile answered, carefully listening to the questions, etc. |

72. **Listening attentively to patient's questions**

Patients expect that the doctor not only listen to their disease with patience and attention, but also listen to their questions with patience and attention. In this regard, assess the role of the doctor by 1 to 4, where 1-completely unsatisfactory and 4-completely satisfactory. Possible observations are as follows: **[If the patient does not have any questions, fill in the ‘Not Applicable’ field.]**

| NA-------1-------2--------3--------4 | | | |
| --- | --- | --- | --- |
| When the patient asked question, the doctor did not show attention and patience through his behavior and words. | When the patient asked question, the doctor showed least attention and patience through his behavior and words. (as for example, at least any of the following issues) | When the patient asked question, the doctor showed some attention and patience through his behavior and words. (as for example, asking question to hear more and any two of the following issues) | When the patient asked question, the doctor showed clear and full attention and patience through his behavior and words. (most or all of the following issues) |
| **Behavior expressing attention and patience might be:** shaking head while listening, looking at the patient, asking question to hear more, the nuances of voice, smiling, some interest revealing words (e.g.,: Well, hmm, etc.) and so on. | | | |

73. **Not using jargon**

One of the most important impediments for patients to understand doctor's advice is the medical terminology (jargon), professional language etc. So, the doctor should avoid such language or explain it if used. In this regard, assess the role of the doctor by 1 to 4, where 1-completely unsatisfactory and 4-completely satisfactory. Possible observations are as follows: **[If the doctor does not say any word to the patient, fill in the ‘Not Applicable’ field.]**

| NA-------1-------2--------3--------4 | | | |
| --- | --- | --- | --- |
| The doctor used many medical terminologies, and did not explain it to the patient. | The doctor used many medical terminologies, and explained some of those to the patient. | The doctor used one or two medical terminologies, and also explained. | The doctor did not use any medical terminology. |
| **Example of medical terms:** pus cell (puj), absorb (digestion), nebulize (providing gas) | | | |

74. **Communicating limitations to the patient at the outset**

In our country patients often become engaged in argument with doctors, the most common reason of it is disproportionate expectations of patients with attainment. So, if there is possibility of such worse situation, then the doctor should inform the patients at the beginning that which services they can get from the doctors and which is not possible to get. In this way patient’s expectations become realistic and it is possible to avoid many unnecessary disputes. In this regard, assess the role of the doctor by 1 to 4, where 1-completely unsatisfactory and 4-completely satisfactory. Possible observations are as follows: **[Fill in the “N/A” field if there are no circumstances for telling about the capabilities or limitations (examples of such type of situation are given below.]**

| NA-------1-------2--------3--------4 | | | |
| --- | --- | --- | --- |
| The doctor did not tell anything about his capabilities and limitations to the patients. As a result, undesirable situation such as tug of war, dispute etc. was created. | The doctor did not tell anything about his capabilities and limitations to the patients. As a result, the patient had a little excitement or dissatisfaction. | The doctor told least about his capabilities or limitations to the patients to avoid such type of situations. | The doctor explained his limitations and capabilities to the patients clearly and became sure that the patients had understood. |
| **Example of telling patients about capabilities and limitations may be:** It might be needed to refer the patient if any complexity arises at the time of caesarian operation; telling patients in advance about delay due to patient’s load and doctor’s limitation; explaining any other reason of delay to the patients; if patient shows impatience (or if there is possibility of showing impatience, such as prescribing costly medicine from outside) because of getting no free medicine (that means if not prescribed), then giving the patients idea about free medicines which are available in the hospital and which are not; telling about the shortage of beds in the hospital; if patient comes with any complex disease (such as, MI, stroke) and if its proper treatment is not available, then informing the patient and making aware about possible consequences; if the doctor goes to anywhere else due to any reason (such as attending patients in emergency room, attending patients at indoor), then informing the patients before going there; if patient’s condition is getting worse, then it should be informed to the patient’s attendants at the beginning and it should be discussed with them etc. | | | |

**Closing Salutation**

75. **Closing salutation by doctor**

Saying good-bye is also as important part of consultation as greetings. In this regard, doctor should tell the summary of consultation, verify whether the patient has understood everything properly, tell the patient how to communicate with the doctor if any problem arises later and say goodbye with smile. In this regard, assess the role of the doctor by 1 to 4, where 1-completely unsatisfactory and 4-completely satisfactory. Possible observations are as follows:

| NA-------1-------2--------3--------4 | | | |
| --- | --- | --- | --- |
| The doctor did not say good-bye; even when patient said goodbye he did not reply. | The doctor hardly said good-bye to the patient.  When patient said goodbye to the doctor, he replied by using gesture (by nutation, eye contact, waving hand etc.). | The doctor said good-bye to the patient but it was not complete or enough.  When patient said good-bye, the doctor replied him verbally. **Examples may be:** saying walaikumassalam; ok, you may go; be well etc. | The doctor said goodbye to the patient properly.  When patients said good-bye, the doctor responded warmly. **As a good practice, following issues might be included:** summarizing the whole consultation; verifying whether the patient has understood everything properly or not; telling the patient how to communicate with the doctor if any problem arises later and saying goodbye with smile (assalamualaikum, khodahafez, be well, etc.) |

76. **Responding to patient's closing salutation**

In our country most of the time patients say goodbye to doctors. Patients expect that doctor would reply. In this regard, assess the role of the doctor by 1 to 4, where 1-completely unsatisfactory and 4-completely satisfactory. Possible observations are as follows: [**Fill in the “N/A” field if patient does not say goodbye.]**

| NA-------1-------2--------3--------4 | | | |
| --- | --- | --- | --- |
| Patient said goodbye but the doctor did not even reply. | When patient said goodbye to the doctor, he replied by using only gesture (by moving neck, eye contact, waving hand, etc.), but did not tell anything verbally. | When patient said goodbye to the doctor, he replied briefly; such as: by moving neck, eye contact, waving hand and along with it replied the salam verbally (walaikumassalam, khodahafez, Allah hafez) | When patient said goodbye, the doctor replied warmly and appropriately and he also said good-bye (khodahafez, be well, etc.). |

77. **Legibility of prescription**

Patients expect that the doctor’s handwriting would be readable so that they can follow the prescription accordingly. Prescription should be easily followable. In this regard, assess the role of the doctor by 1 to 4, where 1-completely unsatisfactory and 4-completely satisfactory. Possible observations are as follows: **[Read the prescriptions. Fill in the “N/A” field if it is not possible to check the prescription.]**

| NA-------1-------2--------3--------4 | | | |
| --- | --- | --- | --- |
| None of the following three features was present. | At least one of the following three features was present. | Two of the following three criteria were present. | All of the following three criteria were present. |
| **Features of a good prescription may be:** The handwriting of the doctor was clear. There were details about treatment in the prescription (such as if which medicine to take and when to take is written in such a way that is readily understandable to the patient). Diet, what can be done, what cannot be done, other advices etc., were also mentioned in the prescription. | | | |

**Throughout Consultation**

78. **Not showing hierarchical difference**

Patients expect that the doctor’s behavior would not show hierarchical difference. In this regard, assess the role of the doctor by 1 to 4, where 1-completely unsatisfactory and 4-completely satisfactory. Possible observations are as follows:

| NA-------1-------2--------3--------4 | | | |
| --- | --- | --- | --- |
| Hierarchical difference was very clearly evident in doctor’s behavior and consultation process.  **Hierarchical difference could be understood by the following issues:** Difference between doctor’s and patient’s chair (The doctor sits on the cushioned chair whose height is slightly more and there is a towel on the backrest of the chair; patients sit on the tool.)(However, if there is no sitting arrangement for the patient in the room, then it might not be due to the insensitivity of the doctor, rather it might be due to faulty management of hospital.); though patients have to enter the doctor’s room by putting off shoes but the doctor enters with shoes; asking the patients to address the doctor as “Sir”; many differences in behavior with patients by his dress up, type of sitting, tone of voice (authoritative tone), and style of speech that means the doctor’s behavior; not looking at patients etc. | Hierarchical difference was quite clearly visualized in doctor’s behavior and consultation process.  **Hierarchical difference could be understood by the following issues:** Difference between doctor’s and patient’s chair (The doctor sits on the cushioned chair whose height is slightly more and there is a towel on the backrest of the chair; however, there were good chairs also for patients)(However, if there is no sitting arrangement for the patient in the room, then it might not be due to the insensitivity of the doctor, rather it might be due to faulty management of hospital.); a little bit differences in behavior with patients by his dress up, type of sitting, tone of voice (authoritative tone), and style of speech that means the doctor’s behavior etc. | Hierarchical difference was not clearly visualized in doctor’s behavior and consultation process. However, initiative expressing equality was also absent.  **Hierarchical difference could be understood by the following issues:** Slight difference between doctor’s and patient’s chair; however, there were good chairs also for patient’s sitting; (However, if there is no sitting arrangement for the patient in the room, then it might not be due to the insensitivity of the doctor, rather it might be due to faulty management of hospital.); however, observing not much difference in doctor’s behavior with patients. | No Hierarchical difference was seen in doctor’s behavior and consultation process; moreover, it was found that the doctor took initiative for showing equality. Symptoms of hierarchical difference mentioned earlier were absent. |

79. **Gender sensitivity**

Doctors should show gender sensitivity and should refrain from gender insensitive behavior. In this regard, assess the role of the doctor by 1 to 4, where 1-completely unsatisfactory and 4-completely satisfactory. Possible observations are as follows: **[If the patient is not a female, fill in the “Not Applicable” field.]**

| NA-------1-------2--------3--------4 | | | |
| --- | --- | --- | --- |
| The doctor clearly showed gender insensitive behavior. | Some (at least one) of doctor's behavior can be regarded as gender insensitive. | The doctor remained away from any kind of gender insensitive behavior. However, he also did not leave any example of gender sensitive behavior. | The doctor remained away from any kind of gender insensitive behavior. Not only that, he did some clear gender sensitive behavior. |
| **Examples of gender insensitive behavior may be:** Making comments to degrade women; avoiding legal assistance related issues to women who are affected by violence; providing poor services to female; making abusive comments to the female for rejecting gender norm; making comment by considering violence against women as usual; blaming the victim; asking male attendant for taking consent about treatment of female patient or advice etc.  **Examples of sensitive behavior to female in context of Bangladesh may be:**  Providing service to female patients on priority basis (especially aged and pregnant); expressing the matter of giving importance to female patients by behavior; maintaining modesty while physical examination; maintaining privacy, etc. | | | |

80. **Interruption during consultation**

Patients do not expect any sort of disturbance (interruption) during consultation. On the basis of interruption assess the consultation within 1 to 4, where 1-completely unsatisfactory and 4-completely satisfactory. Possible observations are as following:

| NA-------1-------2--------3--------4 | | | |
| --- | --- | --- | --- |
| Severe interruption occurred during consultation. **For example:** The doctor called someone over phone; entrance of medical representatives inside(that means allowing them inside by the doctor);calling someone else inside the room by the doctor (that means he did not come intentionally rather the doctor called him or the doctor allowed him). | **In this case examples of interruption may be:** Entrance of any other patient (that means allowing him by the doctor); entrance of any person familiar with the doctor (the doctor did not call him); the doctor left the room for some reason etc. Fill in the field ‘1’ if any of these events occurs more than once. | **In this case examples of interruption may be:** Incoming call in the doctor’s mobile phone (he did not make the call); entrance of clerk or anyone else inside the room during consultation for official purpose; knocking at the door by medical representative (but he did not enter the room); entering any other doctor or nurse inside the room for some reason. Fill in the field ‘2’ if any of these events occurs more than once. | No interruption occurred during consultation. |

81. **Appearance of doctor**

Doctor’s appearance should be neat and clean, tidy and professional. In this regard, assess the role of the doctor by 1 to 4, where 1-completely unsatisfactory and 4-completely satisfactory. Possible observations are as follows:

| NA-------1-------2--------3--------4 | | | |
| --- | --- | --- | --- |
| Two among the following features were less visible in the doctor’s appearance: neat and clean, formal, modest. | The doctor did not wear apron but at least two of the following characteristics were present in his appearance: neat and clean, formal, modest. | The doctor did not wear apron but at least three of the following characteristics were present in his appearance: neat and clean, formal, modest. | The doctor wore apron and at least three of the following characteristics were present in his appearance: neat and clean, formal, modest. |
| **Formal dress may be:** Pant, shirt (Male), sharee, salower-kamiz (female).  **Example of professional dress:** Apron.  **Modest Dress:** According to the social norms of Bangladesh, any dress, which is not offensive, would be considered as modest; in this case, keeping veil or wearing religious dress is not necessary. | | | |

82. **Establishing discipline in consultation room**

Patients expect that the doctor would take initiative to bring back the discipline if there is no discipline inside the doctor’s room (where there is no measure to control patients). In this regard, assess the role of the doctor by 1 to 4, where 1-completely unsatisfactory and 4-completely satisfactory. Possible observations are as follows: **[Fill in the “NA” field if there is discipline already inside the doctor’s room; that means if there is no chance to observe the role of the doctor in establishing discipline]**

| NA-------1-------2--------3--------4 | | | |
| --- | --- | --- | --- |
| There was complete chaos, and the doctor did not take any measure to control it. | The doctor tried to establish discipline (least) in the room because of patient’s request. However, it did not work (that means discipline was not established in the room). | The doctor established discipline in the room because of patient’s request and it was effective (that means discipline was established in the room). | The doctor established discipline in the room spontaneously and it was effective (that means discipline was established in the room).As for example, calling patients one by one according to the ticket or slip. |

83. **Non-verbal communication by doctor**

Patients expect that doctors would do non-verbal communication, such as keeping hand on the body (for assuring the patient), eye contact, etc. In this regard, assess the role of the doctor by 1 to 4, where 1-completely unsatisfactory and 4-completely satisfactory. Possible observations are as follows:

| NA-------1-------2--------3--------4 | | | |
| --- | --- | --- | --- |
| The doctor did not do any non-verbal communication with the patients. | Shaking head while talking, eye contact with the patients, etc. | Shaking head while talking, eye contact with the patients, variation of pitch, smiling face, etc. | Shaking head while talking, eye contact with the patients, variation of pitch, smiling face, holding patient’s hand, keeping hand on the patient’s shoulder, rubbing on the head, sitting lean forward, etc. |

84. **Compassionately touching the patient by doctor**

Patients expect that doctors would touch them. In this regard, assess the role of the doctor by 1 to 4, where 1-completely unsatisfactory and 4-completely satisfactory. Possible observations are as follows:

| NA-------1-------2--------3--------4 | | | |
| --- | --- | --- | --- |
| The doctor did not even touch the patient. | The doctor touched the patient at least once for physical examination or for providing consolation or reliance. | The doctor touched the patient more than once for physical examination or for providing consolation or reliance. | The doctor touched the patient several times for various purposes. As for example, for examining, for providing consolation or reliance. |

85. **Not being involved in illegal activities**

Patients do not want to see the doctor involved in illegal or unethical activity, especially if it is related with their treatment. The doctor should remain away from such type of activity. Assess the role of the doctor for remaining away from visualized immoral activities by 1-4, where 1-completely unsatisfactory and 4-completely satisfactory. Possible observations are as follows: **[Fill in the “N/A” field if the thing could not be observed for any reason.]**

| NA-------1-------2--------3--------4 | | | |
| --- | --- | --- | --- |
| The doctor was involved with various illegal activities. | The doctor was involved with at least one illegal activity. | The doctor was not directly involved with any illegal activity. However, such kind of activities happened in front of him and he remained silence that means he had tacit support. Examples: ignoring presence of brokers (inside the room), behavior with medical representatives seen to be over treated etc. | The doctor was not involved with any illegal activity and he was not seen to support any such activities either directly or indirectly. |
| **Examples of such illegal activities may be** taking money from patients against free services; bringing patients with the help of brokers; presence of brokers around the chamber (Doctors did not take any step to stop this); collusion with diagnostic centers. Accepting gift from medical representative (and prescribing medicine of that company); taking advantage from brokers (utilizing in hospital, utilizing for personal work) and so on. | | | |

86. **Sense of humor**

Patients expect that the doctor would have sense of humor and would provide treatment by becoming easy with patients using his sense of humor. In this regard, assess the role of the doctor by 1 to 4, where 1-completely unsatisfactory and 4-completely satisfactory. Possible observations are as follows:

| NA-------1-------2--------3--------4 | | | |
| --- | --- | --- | --- |
| The doctor was in serious mood. He did not have any kind of humor. | The doctor was smiling or he had some humor. | The doctor was smiling and he had some humor. | The doctor was smiling all the time and made the patient feel comfortable by joking. |
| Examples of humor may be **(examples from my participant observation):** When a young lady asked for vitamin syrup, the doctor replied, “Leave these baby foods”. After doing ultra sonogram the doctor told the child patient, “Go and run now”. During ultra sonogram the doctor told a female patient, “I see that you have eaten very well at noon”. | | | |

87. **Relaxedness and confidence**

Patients expect that the doctor would be relaxed and confident. There would be no tension and anxiety in his behavior. In this regard, assess the role of the doctor by 1 to 4, where 1-completely unsatisfactory and 4-completely satisfactory. Possible observations are as follows:

| NA-------1-------2--------3--------4 | | | |
| --- | --- | --- | --- |
| Stress and anxiety were expressed in doctor’s behavior. | Even though the doctor’s behavior did not show stress and anxiety, he did not look relaxed and confident. | The doctor’s behavior looked slightly relaxed and confident. | The doctor was fully relaxed and confident. |
| **Stress and anxiety expressing behavior may be:** restless movement of hand, feet, pen, and nervous laughter.  **Confidence expressing behavior may be:** The doctor would say, “You will get well after taking this.” | | | |

88. Patients expect that the doctor would give them enough time. For assessing the role of the doctor in this regard record the consultation time (in seconds) using a stopwatch.

89. Patients expect that the doctor would not consult with more than a certain number of patients every day. For assessing the role of the doctor in this regard record the number of patients he attended in a day in public/private sector (which is applicable) based on the answer to the question number 22.

**90. Questions for 11th Patient**

**Instruction to the observer:** When the patient leaves the room, please leave the room along with the patient and record the following information.

91. Age of the patient

92. Gender of the patient

93. Educational background of the patient
